# Protective efficacy of an attenuated *Mtb* ΔLprG vaccine in mice

**DOI:** 10.1371/journal.ppat.1009096

**Published:** 2020-12-14

**Authors:** Amanda J. Martinot, Eryn Blass, Jingyou Yu, Malika Aid, Shant H. Mahrokhian, Sara B. Cohen, Courtney R. Plumlee, Rafael A. Larocca, Noman Siddiqi, Shoko Wakabayashi, Michelle Gardner, Rebecca Audette, Anne Devorak, Kevin B. Urdahl, Eric J. Rubin, Dan H. Barouch

**Affiliations:** 1 Center for Virology and Vaccine Research, Beth Israel Deaconess Medical Center, Harvard Medical School, Boston, Massachusetts, United States of America; 2 Department of Infectious Diseases and Global Health, Tufts University Cummings School of Veterinary Medicine, North Grafton, Massachusetts, United States of America; 3 Department of Immunology, Seattle Children’s Research Institute, Seattle, Washington, United States of America; 4 Department of Immunology and Infectious Diseases, Harvard School of Public Health, Boston, Massachusetts, United States of America; 5 Departments of Pediatrics and Immunology, University of Washington, Seattle, Washington, United States of America; 6 Ragon Institute of MGH, MIT, and Harvard, Cambridge, Massachusetts, United States of America; New Jersey Medical School, UNITED STATES

## Abstract

Bacille Calmette-Guerin (BCG), an attenuated whole cell vaccine based on *Mycobacterium bovis*, is the only licensed vaccine against *Mycobacterium tuberculosis* (*Mtb*), but its efficacy is suboptimal and it fails to protect against pulmonary tuberculosis. We previously reported that *Mtb* lacking the virulence genes *lprG* and *rv1410c (*ΔLprG) was highly attenuated in immune deficient mice. In this study, we show that attenuated ΔLprG *Mtb* protects C57BL/6J, Balb/cJ, and C3HeB/FeJ mice against *Mtb* challenge and is as attenuated as BCG in SCID mice. In C3HeB/FeJ mice, ΔLprG vaccination resulted in innate peripheral cytokine production and induced high polyclonal PPD-specific cytokine-secreting CD4^+^ T lymphocytes in peripheral blood. The ΔLprG vaccine afforded protective efficacy in the lungs of C3H/FeJ mice following both H37Rv and Erdman aerosolized *Mtb* challenges. Vaccine efficacy correlated with antigen-specific PD-1-negative CD4^+^ T lymphocytes as well as with serum IL-17 levels after vaccination. We hypothesize that induction of Th17 cells in lung is critical for vaccine protection, and we show a serum cytokine biomarker for IL-17 shortly after vaccination may predict protective efficacy.

## Author summary

Many successful vaccines are based on attenuated human pathogens. The only licensed tuberculosis vaccine, BCG, is based on an attenuated version of live whole cell *Mycobacterium bovis*, the causative agent of tuberculosis (TB) in cattle. Advantages to using attenuated pathogens as vaccines include a broad antigen composition including proteins, lipids, carbohydrates and other molecules that can induce durable immune responses sometimes lasting decades. Here we test an attenuated *Mycobacterium tuberculosis (Mtb)*, the causative agent of human TB, that lacks a key virulence factor as an alternative whole cell vaccine in mice. Attenuated *Mtb* lacking a key virulence protein, LprG, is immunogenic and protects mice against *Mtb* challenge. The LprG whole cell vaccine is protective in mice that develop lung pathology more similar to what is described in human TB and the LprG vaccine induces a key cytokine, IL-17, thought to be important for vaccine protection, in the peripheral blood early after vaccination. Together these data support the continued development of attenuated TB as a potential vaccine candidate. Furthermore our data suggests that serum IL-17 should be explored as a potential biomarker for vaccine efficacy in preclinical animal models.

## Introduction

BCG is currently the only approved TB vaccine for use in humans. While it protects against childhood TB meningitis, it provides only limited protection in adulthood [1]. Several BCG strains are available worldwide [2] and all are derived from attenuated strain *Mycobacterium bovis*, the mycobacterial agent of bovine tuberculosis. BCG attenuation is based on the loss of key virulence factors such as ESAT-6 and CFP-10 [3]. BCG vaccination is almost universal in high burden TB regions of the world. Therefore, any novel vaccination regimen should demonstrate, at a minimum, equivalent protective efficacy as BCG and ideally would surpass the protective benefit provided by BCG vaccination. Recent studies have suggested that boosting BCG with secondary doses of BCG or novel adjuvanted vaccines such as M72/AS01_E_ may improve TB disease outcomes [4,5]. Data in non-human primates (NHP) showing the protective efficacy of prior *Mtb* infection on preventing subsequent acquisition of *Mtb* after re-exposure [6] coupled with recent clinical safety data on the use of attenuated whole cell *Mtb* vaccines suggest that the continued development of attenuated *Mtb* as TB vaccines is warranted [7].

Mycobacteria are characterized by a thick, lipid-rich cell wall composed of covalently bound peptidoglycan linked to arabinogalactan and mycolic acids [8,9]. Numerous non-covalently bound antigenic lipids are intercalated within the mycolic acid layer, many of which have been shown to play important roles in virulence and the host-pathogen interaction with *Mtb*. *Mtb* devotes a large portion of its genome to lipid synthesis and lipid export to maintain its elaborate cell wall structure. The genes *rv1411c-rv1410c*, encoding a lipoprotein (LprG) and transmembrane efflux pump (Rv1410), have been shown to be conditionally essential for *in vivo* survival in the murine model. Our previous work characterized this operon as a lipid transporter [10], disruption of which altered the lipid content of the *Mtb* cell wall and its metabolic state, leading to marked attenuation in both immunocompetent (C57BL/6J) and immunodeficient (*Rag -/-*, SCID, *phox -/-*, and *infr -/-)* mice [11].

The LprG lipoprotein is a potent TLR2 agonist [12] and has been hypothesized to play a role in host immune evasion by decreasing antigen presentation by macrophages *in vitro* [13]. LprG also binds immunomodulatory lipids that can prevent phagolysosome fusion, which may impact downstream MHC class II antigen processing [14,15]. Given the profound attenuation observed with loss of the LprG-Rv1410 operon in immunodeficient mice and the immunomodulatory role of LprG, we hypothesized that deletion of the LprG-Rv1410 locus may result in an improved whole cell *Mtb* vaccine.

## Results

### The ΔLprG vaccine protects against Mtb challenge in mice and has a comparable attenuation to BCG

**Fig 1:**
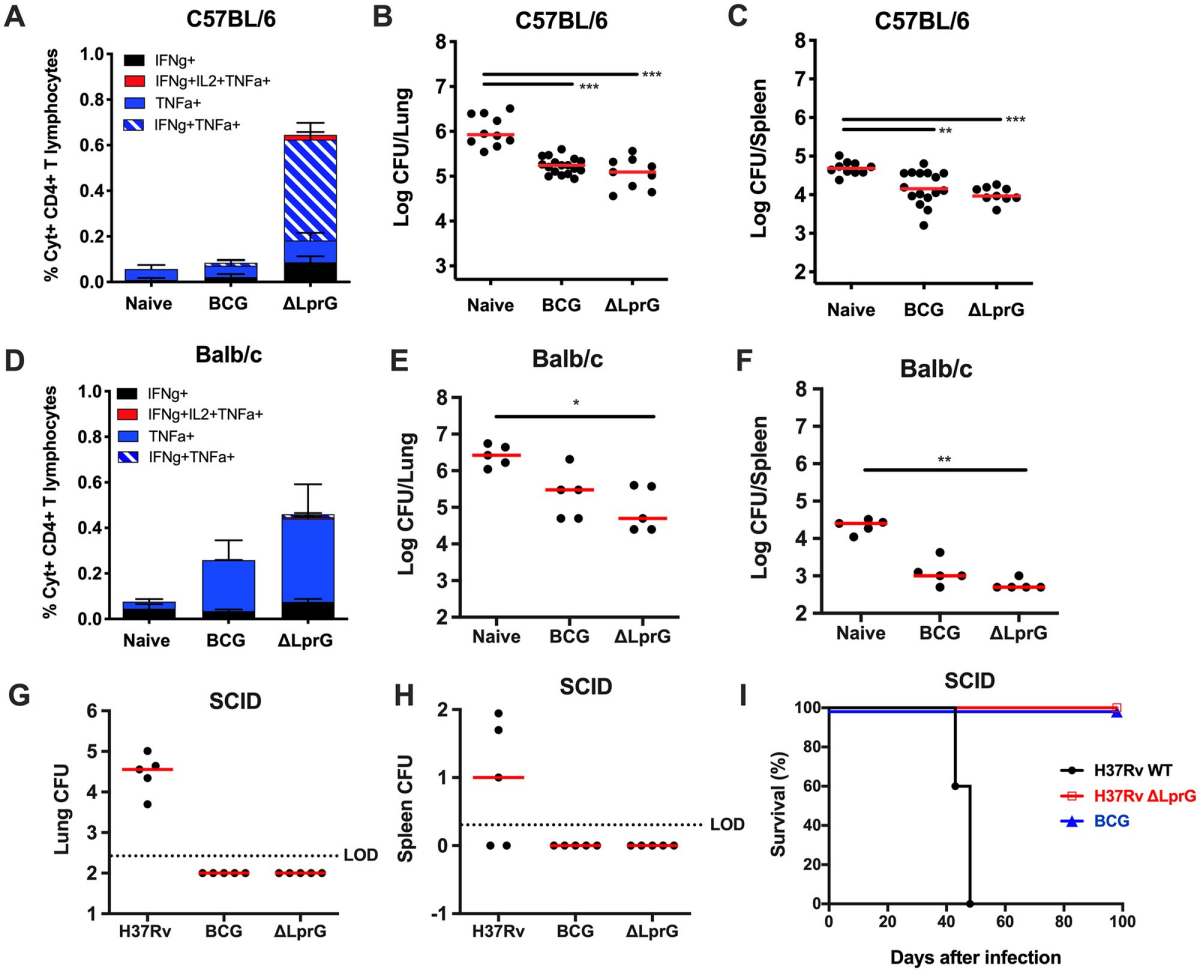
Protective efficacy of ΔLprG and BCG vaccines in C57BL/6J and Balb/cJ mice and attenuation in SCID mice. C57BL/6J or Balb/cJ mice were vaccinated with 100uL of O.D. 1.0 log-phase culture of either BCG Serum Statens Institute (SSI) or H37RvΔ*lprG-rv1410c* (ΔLprG) subcutaneously in the left flank. Peripheral blood mononuclear cells (PBMC) were collected 2 weeks post-vaccination. Percent cytokine positive (%Cyt^+^) antigen-specific T lymphocyte responses are shown as measured by intracellular cytokine staining (ICS) in CD4^+^ CD44^+^ T lymphocytes following stimulation with purified protein derivative (PPD, Synbiotic Tuberculin OT). Mice were aerosol-challenged with 75 CFU of *Mtb H37Rv*. Colony-forming units (CFU) were counted from lung or spleen at week 4 following challenge; C57BL/6 **(A-C) and** Balb/cJ **(D-F)** mice. SCID mice received approximately 75 CFU of aerosolized *Mtb H37Rv*, BCG SSI, or ΔLprG. CFU from lung **(G)** or spleen **(H)** of SCID mice 4 weeks post-infection. **I)** Survival curve from **(G,H)** showing survival of mice receiving aerosolized ΔLprG and BCG vaccines out to 100 days. Kruskall-Wallis with Dunn’s correction for multiple comparisons; * p<0.05; ** p<0.01; *** p<0.001; **** p<0.0001. Red bar = median. Data is one representative experiment out of three with 5–15 mice per group for C57BL/6J mice. Balb/cJ and SCID challenges were performed once with 5 mice per group.

We wanted to evaluate the potential use of the previously characterized H37Rv*ΔlprG-rv1410* (ΔLprG) deletion strain [11] as an alternative whole cell vaccine to BCG. We determined that ΔLprG induced Ag-specific T cells in peripheral blood of both C57BL/6J and Balb/cJ mice and tested the protective efficacy of the ΔLprG mutant as a vaccine strain in these backgrounds (Fig 1A–1F). Both ΔLprG and BCG vaccinated C57BL/6 (Fig 1A–1C) and Balb/cJ mice (Fig 1D and 1F) demonstrated immunogenicity and reduced bacterial loads as compared to non-vaccinated mice (Naïve) after *Mtb* challenge. We have previously published that the ΔLprG deletion strain was significantly attenuated in SCID mice as compared to WT and complemented mice after IV inoculation (Martinot et al., 2016). We further confirmed the attenuation phenotype with an aerosol administration of the ΔLprG vaccine in SCID mice, which showed a similar attenuation profile to BCG (Fig 1G and 1H). All BCG and ΔLprG vaccinated mice survived >100 days, whereas all *Mtb* infected mice died by day 50 (Fig 1I).

### ΔLprG vaccination leads to enhanced immunogenicity and protection in mice that develop necrotizing granulomas

**Fig 2:**
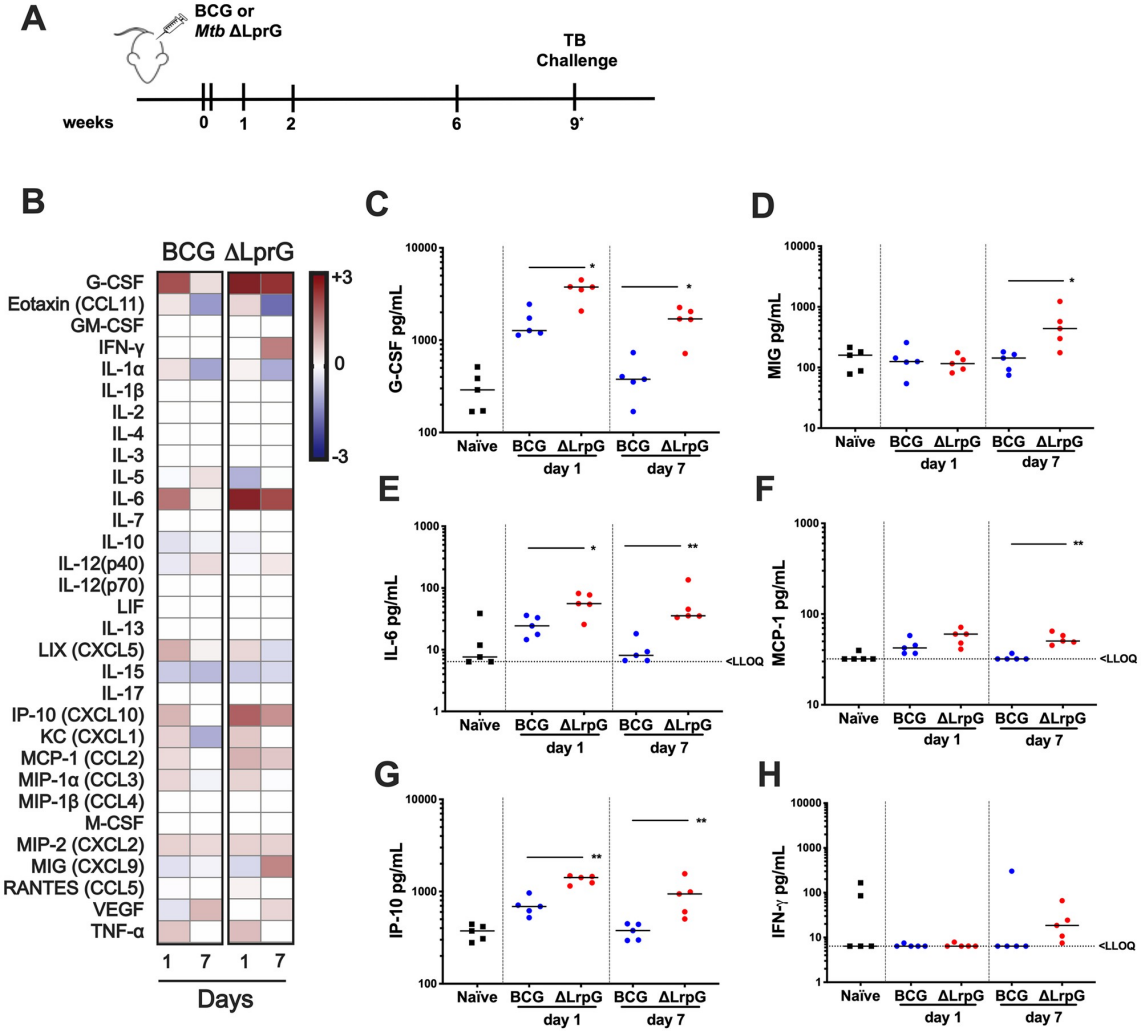
Induction of pro-inflammatory cytokines by ΔLprG and BCG vaccines in C3HeB/FeJ mice. **A)** Vaccination regimen. C3HeB/FeJ mice were vaccinated with 100uL of O.D. 1.0 log-phase culture of either BCG Serum Statens Institute (SSI) or H37RvΔ*lprG-rv1410c* (ΔLprG) subcutaneously in the left flank. Serum for Luminex cytokine analysis was collected on days 1 and 7 after vaccination. PBMC were collected at weeks 2, 6, and 9 post-vaccination. **B)** Heat-map showing median log2 fold-change of serum cytokine levels as compared to naïve mice in BCG and ΔLprG vaccinated mice on days 1 and 7. **C-H)** Individual cytokine levels in serum on days 1 and 7; bar represents median values;* p<0.05; ** p<0.01, Mann-Whitney U test (BCG vs. ΔLprG). LLOQ represents lower limit of quantification. Luminex assays were performed twice with 5–8 mice per group. Data is representative of individual experiments.

We were interested in studying the effect of attenuated whole cell vaccines on the structure and function of TB granulomas. We reasoned that vaccination studies in mice may fail to predict vaccine efficacy in humans in part due to the fact that common mouse models used in TB vaccine research do not recapitulate classic TB pathology observed in humans. Therefore, we tested the immunogenicity of BCG and ΔLprG vaccines in C3HeB/FeJ mice, which develop necrotizing granulomas that are similar to human TB granulomas. C3HeB/FeJ mice have a genetic susceptibility locus *sst1* that renders them highly susceptible to tuberculosis disease [16], and they develop lesions with central necrosis characterized by neutrophilic infiltrates that can develop into caseous and hypoxic lesions over time [17,18]. Furthermore, we wanted to assess vaccine immunogenicity longitudinally in peripheral blood of mice. C3HeB/FeJ mice were vaccinated by the subcutaneous route with 2x10^7^ colony-forming units (CFU) of either BCG or ΔLprG, formulated in phosphate-buffered saline with tyloxapol and glycerol [19]. Serum was collected for Luminex analysis of cytokine and chemokine responses on days 0, 1, and 7 following vaccination, and PBMC were isolated at weeks 2, 6, and 9 (Fig 2A). ΔLprG vaccination resulted in greater levels of serum inflammatory cytokines G-CSF (p = 0.0159), IL-6 (p = 0.0317), and IP-10 (p = 0.0079) than BCG on day 1 and day 7 (p = 0.0159, p = 0.0079, p = 0.0079, respectively) following vaccination (Fig 2B–2H). ΔLprG vaccination also led to greater serum MIG (p = 0.0159) and MCP-1 (p = 0.0079) than BCG on day 7 (Fig 2D and 2F).

**Fig 3:**
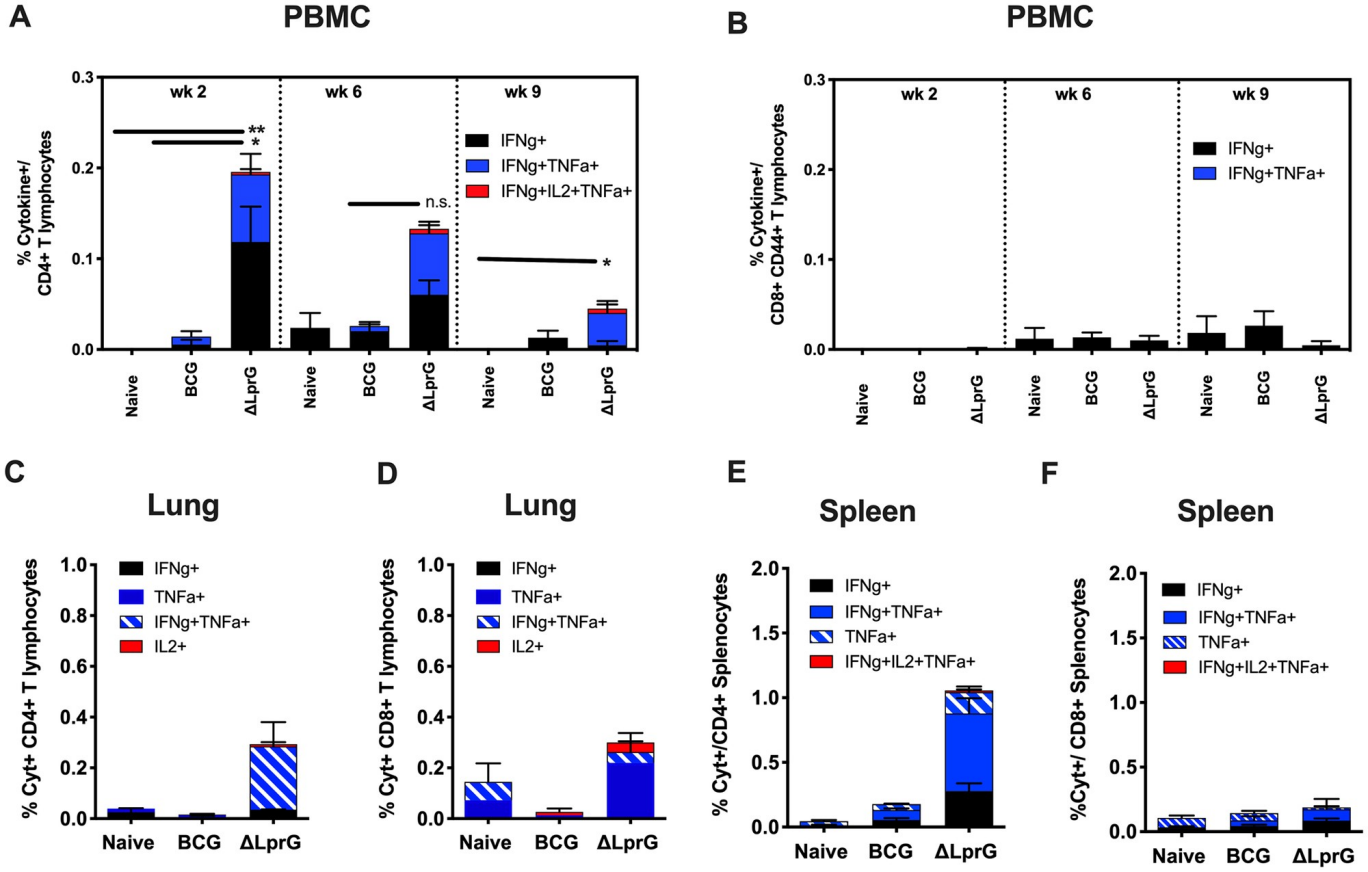
Immunogenecity of BCG and ΔLprG vaccines in C3HeB/FeJ mice. PBMC were collected at week 2, 6, and 9 following vaccination (**A,B**). Lung leukocytes (**C,D**) and splenocytes (**E,F**) were collected 2 weeks following vaccination. Percent cytokine positive (%Cyt^+^) antigen-specific T lymphocyte responses as measured by intracellular cytokine staining (ICS) in CD4^+^ (**A,C,E**) and CD8^+^ (**B,D,F**) CD44^+^ T lymphocytes following stimulation of PBMC, lung leukocytes, or splenocytes with purified protein derivative (PPD, Synbiotic Tuberculin OT). Percentages reflect subsets of cytokine secreting cell populations from Boolean analysis (FlowJo v10) of all possible cytokine combinations (IFNγ, TNF-α, IL-2, IL-17A, and IL-10); * p<0.05; ** p<0.01, Kruskall-Wallis for total % Cyt+ cells with Dunn’s corrections for multiple comparisons. Data is representative of individual experiments. PBMC ICS was performed 4 times with 5–10 animals per vaccine group. Lung leukocytes and splenocyte analyses were performed once with 5–10 animals per group. Data presented is from two different replicates.

We next evaluated Ag-specific T lymphocyte responses in peripheral blood. ESAT-6, Ag85B, and TB10.4 are thought to potentially play a role in both innate immune signaling and protective immunity to TB [3,20,21]. Ag85B and ESAT-6 specific CD4+ T lymphocytes were detected in C57BL/6J mice exposed to *Mtb* lacking the LprG-Rv1410 operon, although at significantly lower levels than those induced by virulent *Mtb*, presumably due to the lack of replication of the mutant *Mtb* as compared to wild-type H37Rv (S1 Fig). On the contrary, C3HeB/FeJ mice are H2-k restricted, and Ag85B and ESAT-6 specific responses were below the limit of detection by intracellular cytokine staining. Therefore, PBMC were stimulated with purified protein derivative (PPD; Tuberculin OT, Synbiotic) and were assessed by ICS assays. Higher IFN-γ-, TNF-α-, and IL-2-secreting CD4^+^ T cell responses were observed in the blood of ΔLprG vaccinated mice as compared to BCG vaccinated mice at week 2 following vaccination (Fig 3A; p = 0.004), and these differences persisted at week 6 and week 9. In contrast, Ag-specific CD8^+^ T cell responses were comparable between ΔLprG and BCG vaccinated mice (Fig 3B) in blood. ΔLprG mice also demonstrated increased numbers of Ag-specific CD4+ T lymphocytes in lung (Fig 3C) and spleen (Fig 3E) 2 weeks post-vaccination. Similar to blood, minimal differences were noted in lung and splenic CD8+T lymphocyte populations post-vaccination (Fig 3D and 3F).

At week 9, mice were challenged by the aerosolized route with 75 colony-forming units (CFU) of *Mtb* H37Rv and were sacrificed at week 4 following challenge to assess protective efficacy and immune correlates of protection. Prior to tissue harvest, lungs were perfused with phosphate-buffered saline (PBS) to clear peripheral erythrocytes and leukocytes from the pulmonary vasculature. Lungs were collected and homogenized, and pulmonary T cells were purified and stimulated with PPD and analyzed by ICS assays for IFN-γ, TNF-α, IL-2, IL-17, and IL-10.

**Fig 4:**
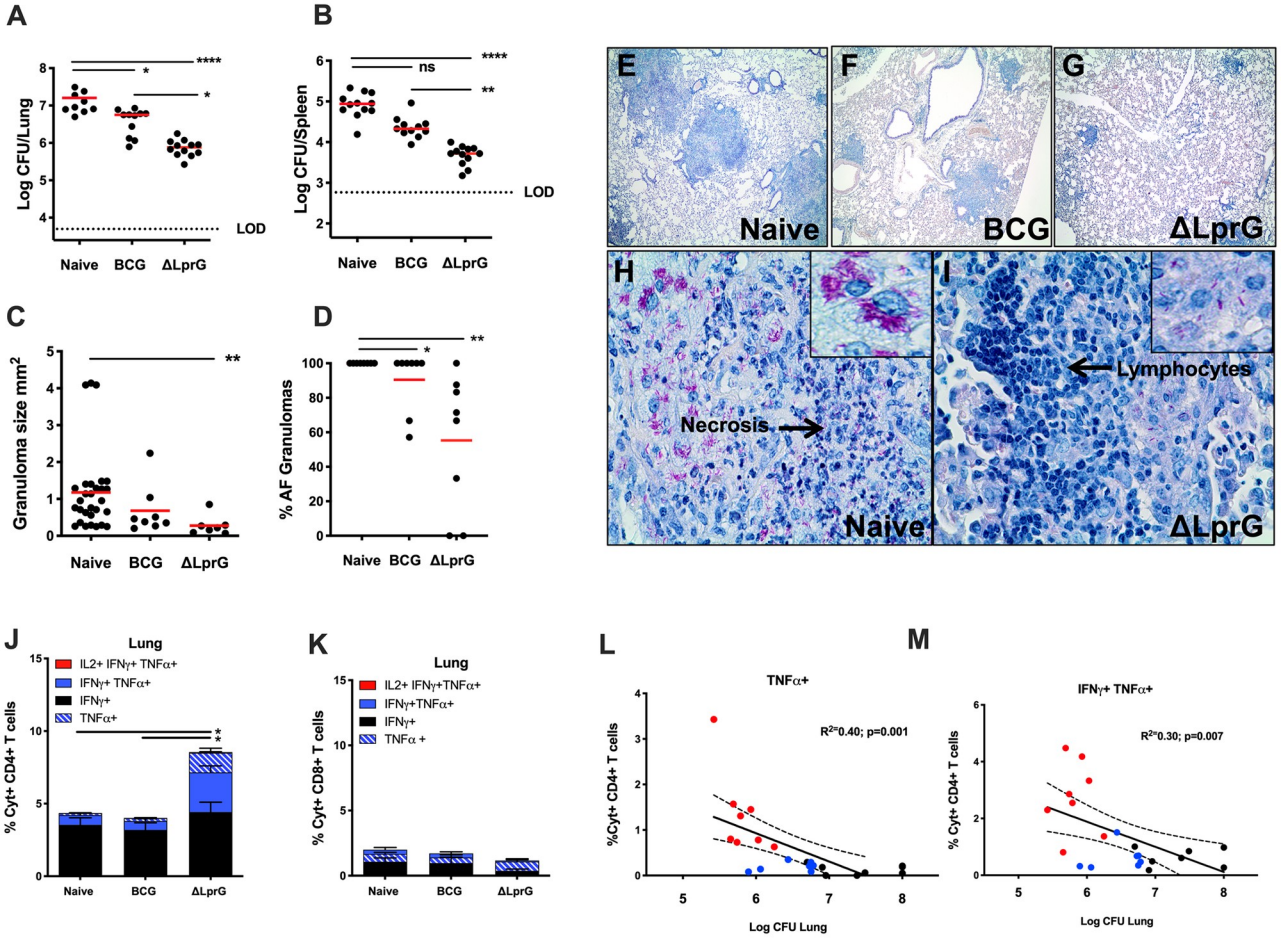
Protective efficacy of ΔLprG and BCG vaccines against *Mtb* challenge in C3HeB/FeJ mice. Colony-forming units (CFU) from **A)** lung or **B)** spleen at week 4 following challenge with 75 CFU of aerosolized *Mtb H37Rv*. Histopathology on lungs from naïve and vaccinated C3HeB/FeJ mice following aerosol *Mtb* challenge showing **C)** individual granuloma size (mm^2^) in lungs from mice at week 4 following challenge and **D)** percent of granulomas that contain visible acid-fast organisms. Dots represent individual animals (**C**) and individual granuloma measurements (**D**). Kruskall-Wallis with Dunn’s correction for multiple comparisons; * p<0.05; ** p<0.01; *** p<0.001; **** p<0.0001. Red bar = median. Representative images of Ziehl-Neelsen acid-fast staining in affected lung from **E)** Naïve **F)** BCG vaccinated and **G)** ΔLprG vaccinated mice, 4 weeks post-*Mtb* aerosol challenge, at 100x magnification. Representative images of Ziehl-Neelsen acid-fast staining in affected lung from **H)** Naïve and **I)** ΔLprG vaccinated mice, 4 weeks post-*Mtb* challenge, at 400x magnification. Percent cytokine positive (%Cyt^+^) antigen-specific T lymphocyte responses as measured by intracellular cytokine staining (ICS) following stimulation with PPD (Synbiotic; Tuberculin OT) in **J)** CD4^+^ or **K)** CD8^+^ CD44^+^ T lymphocytes isolated from lungs of C3HeB/FeJ mice at week 4 following aerosol *Mtb* challenge; * p<0.05, Kruskall-Wallis with Dunn’s corrections for multiple comparisons. Pearson’s correlation (R^2^) of **L)** TNFα^+^ and **M)** IFNγ^+^ TNFα^+^ CD44^+^ CD4^+^ T cells in lung post- *Mtb* challenge with Log10 CFU in lung following challenge. Protection data **(A-D)** is representative of 3 individual challenge experiments with 8–10 mice per group. Lung leukocyte levels post-challenge and CFU correlation analyses **(J-M)** are representative of 2 independent *Mtb* challenges with 8–10 mice per group. Red = ΔLprG-vaccinated; Blue = BCG-vaccinated; Black = Naïve mice.

C3HeB/FeJ mice vaccinated with the ΔLprG vaccine demonstrated a median 1.3 log_10_ reduction in bacterial CFU in lung (Fig 4A) and a 1.2 median log_10_ reduction in bacterial CFU in spleen (Fig 4B) as compared with unvaccinated (Naïve) mice. Although the ΔLprG vaccine resulted in equivalent protection to BCG in C57BL/6J and Balb/cJ mice, the ΔLprG vaccine showed better protection than BCG in C3HeB/FeJ mice with a median 0.9 log_10_ greater reduction in CFU in lung (Fig 4A; p<0.05). BCG afforded less protection in C3HeB/FeJ mice as compared with C57BL/6J and Balb/cJ mice (Fig 1). Dose finding studies in C57BL/6J mice suggested that vaccine dose was unlikely to contribute to the improved protective efficacy seen in C3HeB/FeJ mice vaccinated with the ΔLprG vaccine, since 1 log lower vaccine dose (10^6^ CFU versus 10^7^ CFU) showed only minimal differences in protective efficacy for BCG or ΔLprG in C57BL/6J mice (S2 Fig).

At necropsy, ΔLprG and BCG vaccinated mice had fewer and smaller granulomas as compared to naïve mice (Fig 4C), but ΔLprG vaccinated animals had significantly fewer granulomas with acid-fast bacteria (Fig 4D), consistent with the overall decreased bacterial burden compared to lungs from BCG vaccinated and unvaccinated animals. Unvaccinated mice had multiple granulomas >1 mm^2^ in area (Fig 4C and 4E) with neutrophilic infiltrates and necrosis (Fig 4E), as compared to BCG and ΔLprG vaccinated mice (Fig 4F and 4G). Pathology in non-vaccinated mice was characterized by multibacillary proliferation of acid-fast bacteria within macrophages (Fig 4H, inset), whereas ΔLprG vaccination was associated with formation of granulomas with few to no acid-fast bacteria surrounded by infiltrates of lymphocytes (Fig 4I, inset).

ΔLprG vaccinated mice showed greater Ag-specific CD4^+^ T cell responses in lung than did BCG vaccinated mice and unvaccinated animals at necropsy post-*Mtb* challenge (Fig 4J; p = 0.027 and p = 0.013, respectively). In contrast, no differences were observed in Ag-specific CD8^+^ T cell responses in lung post-challenge across groups (Fig 4K). CD4^+^ and CD8^+^ T cells responses in spleen post-challenge did not substantially differ between groups (S3 Fig). These data suggest that induction of CD4^+^ T lymphocytes in lungs of ΔLprG vaccinated mice may have contributed to the improved protection in C3HeB/FeJ mice. Indeed, reduction in bacterial CFU in lung correlated with both TNF-α- (Fig 4L; p = 0.001) and IFN-γ/TNF-α-secreting CD4^+^ T cells in lung post-challenge (Fig 4M; p = 0.007).

### ΔLprG vaccination is associated with decreased PD-1 expression on antigen specific T cells and correlates with improved bacterial control after Mtb challenge

**Fig 5:**
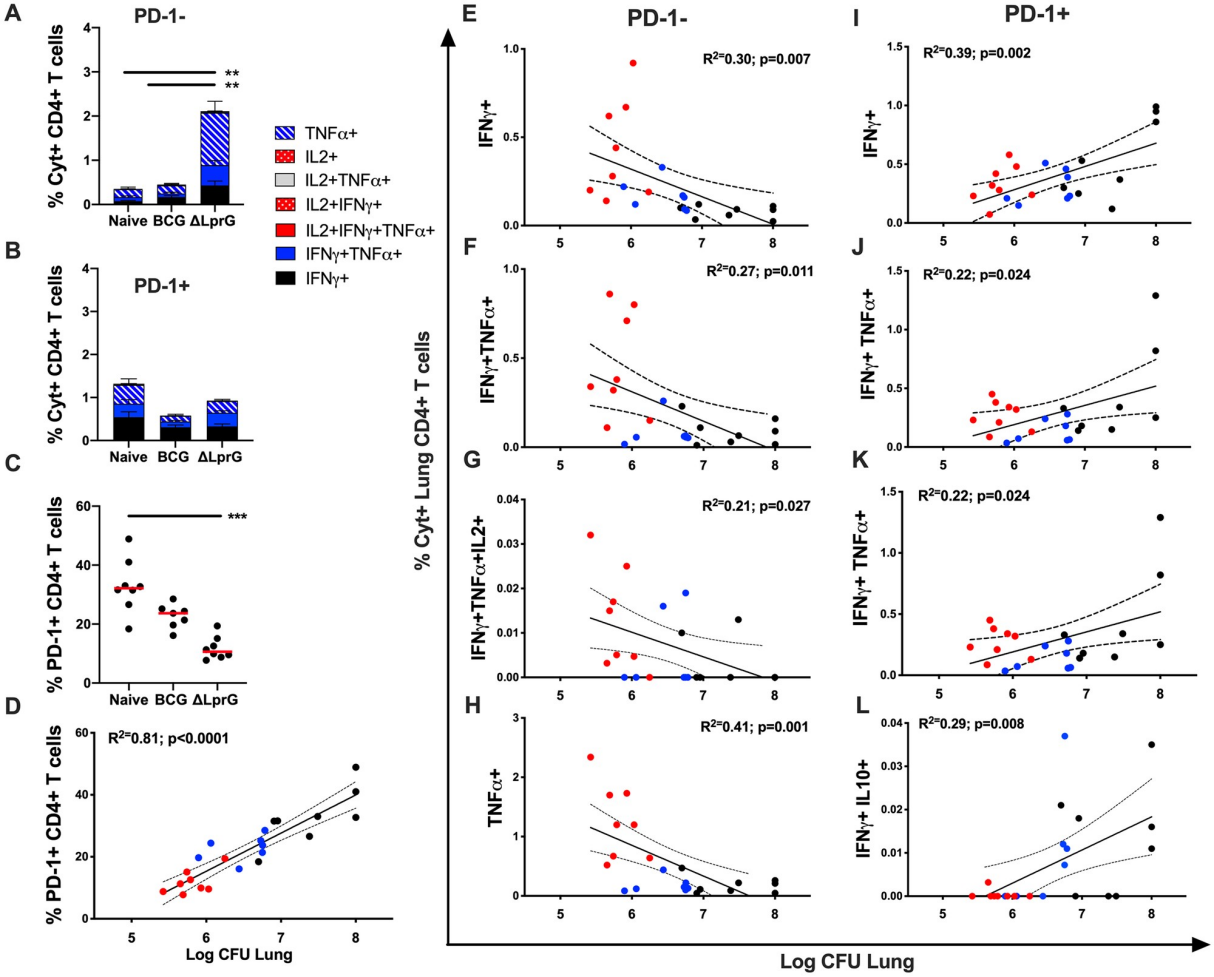
Cellular immune responses in C3HeB/FeJ lung post-*Mtb* challenge in BCG and ΔLprG vaccinated mice. Percent **A)** PD-1-negative (PD-1^-^) and **B)** PD-1-positive (PD-1^+^) cytokine-positive (Cyt^+^) CD4^+^ T cells in lung as measured by intracellular cytokine-staining after stimulation with PPD (Synbiotic; Tuberculin OT) from Naïve and vaccinated mice 4 weeks post-*Mtb* challenge. **C)** Percent PD-1^+^ CD44^+^ CD4^+^ cytokine-negative T lymphocytes. Kruskall-Wallis with Dunn’s corrections for multiple comparisons; * p<0.05; ** p<0.01; *** p<0.001. Red bar = Median. **D)** Pearson’s correlation (R^2^) of percent PD-1^+^ cytokine-negative CD44^+^ CD4^+^ T lymphocytes with CFU in lung following challenge. Pearson’s correlation of PD-1- Cyt^+^ CD44^+^ CD4^+^ T lymphocytes **(E-H)** and PD-1^+^ CD44^+^ CD4^+^ T lymphocytes **(I-L)** with CFU in lung following challenge. **(D-L)** Red = ΔLprG-vaccinated; Blue = BCG-vaccinated; Black = Naïve mice. Data representative of 2 independent experiments with 5–8 mice per group. See also S4 Fig.

Waning of BCG-induced immunity in humans has been reported to be associated with functionally exhausted effector T-lymphocytes [22,23]. PD-1 expression has also been linked to chronic antigenic stimulation and T cell exhaustion during *Mtb* infection [24]. We therefore assessed PD-1 expression on Ag-specific cytokine-secreting (Cyt+) CD4^+^ T lymphocytes from lungs of mice at week 4 following *Mtb* challenge. ΔLprG vaccination resulted in higher PD-1-negative cytokine-secreting Ag-specific CD4^+^ T cell responses in lung than did BCG vaccination or no vaccination post-challenge (Fig 5A; p = 0.002 to p = 0.006; S4 Fig). In contrast, PD-1-positive cytokine-secreting Ag-specific CD4^+^ T cell responses in lung did not differ across groups (Fig 5B) despite variation in bacterial burden across groups (Fig 4A). Prior studies have shown that cells upregulate PD-1 as antigen-experienced cells transition from a central memory to effector state [25]. PD-1-positive cytokine-negative T lymphocytes may represent exhausted T cells [26]. ΔLprG vaccination was associated with significantly lower percentages of cytokine-negative PD-1-positive T cells in lung, reflecting reduced bacterial burden (Fig 5C) and cytokine-negative PD-1-positive CD4^+^ T-lymphocytes in lung correlated with bacterial CFU in lung following challenge (Fig 5D; p<0.0001). Among cytokine-secreting Ag-specific CD4+ T cell subsets, PD-1-negative Ag-specific CD4^+^ T cells in lung correlated with decreased bacterial burden (Fig 5E–5H), whereas PD-1-positive Ag-specific CD4^+^ T cells in lung correlated with increased bacterial burden (Fig 5I–5L), suggesting the importance of PD-1-negative memory T cells for protection against *Mtb* [26].

### ΔLprG vaccine efficacy correlates with serum IL-17A levels post-vaccination in C3Heb/FeJ mice

**Fig 6:**
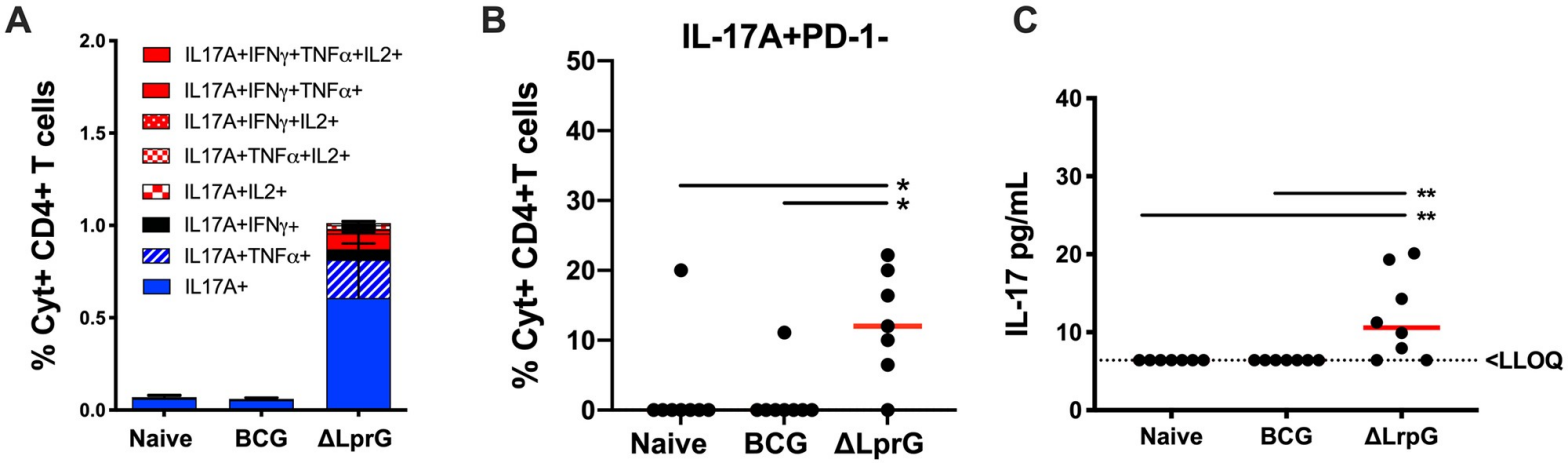
Lung Th17 cells and serum IL-17 induced by ΔLprG and BCG vaccines. **A)** Percent cytokine positive (Cyt+) Ag-specific IL-17A^+^ CD44^+^ CD4^+^ T cells in lung at week 4 after *Mtb* challenge as measured by intracellular cytokine staining (ICS) post-stimulation with PPD (Synbiotic; Tuberculin OT). **B)** Percent total IL17A^+^ PD-1^-^ CD44^+^ CD4^+^ T lymphocytes in lung at week 4 following challenge. Kruskall-Wallis with Dunn’s corrections for multiple comparisons; * p<0.05. Red bar = median. **C)** Serum IL-17 at week 2 after vaccination via Luminex. Kruskall-Wallis with Dunn’s corrections for multiple comparisons;* p<0.05; ** p<0.01. Red bar = median. LLOQ = Lower Limit of Quantification. Lung leukocyte Th17 data and Luminex data representative of 2 independent experiments with 5–8 mice per group. See also S1 Table.

The efficacy of BCG has also been hypothesized to involve IL-17 secreting Ag-specific CD4^+^ T cells (Th17) cells in lung [27,28], and expansion of Th17 cells in lung has been linked to improved vaccine-outcomes for experimental TB vaccines [29,30]. Genome-wide association studies have also linked polymorphisms in IL-17 regulatory genes to poor TB clinical outcomes in patients [31,32]. IL-17+ cells were below the limit of detection in lung and spleen pre-challenge (post-vaccination), and we therefore assessed the expansion of polyfunctional IL-17 secreting CD4+ T cells across vaccine groups following *Mtb* challenge. IL-17A-secreting Ag-specific CD4^+^ T cell populations were increased in ΔLprG vaccinated mice post-challenge compared with naïve or BCG vaccinated mice (Fig 6A). Moreover, ΔLprG vaccinated mice demonstrated significantly higher PD-1-negative IL-17A-secreting CD4^+^ T cells in lung compared to naïve and BCG vaccinated mice (Fig 6B, p = 0.013 and 0.020, respectively).

**Fig 7:**
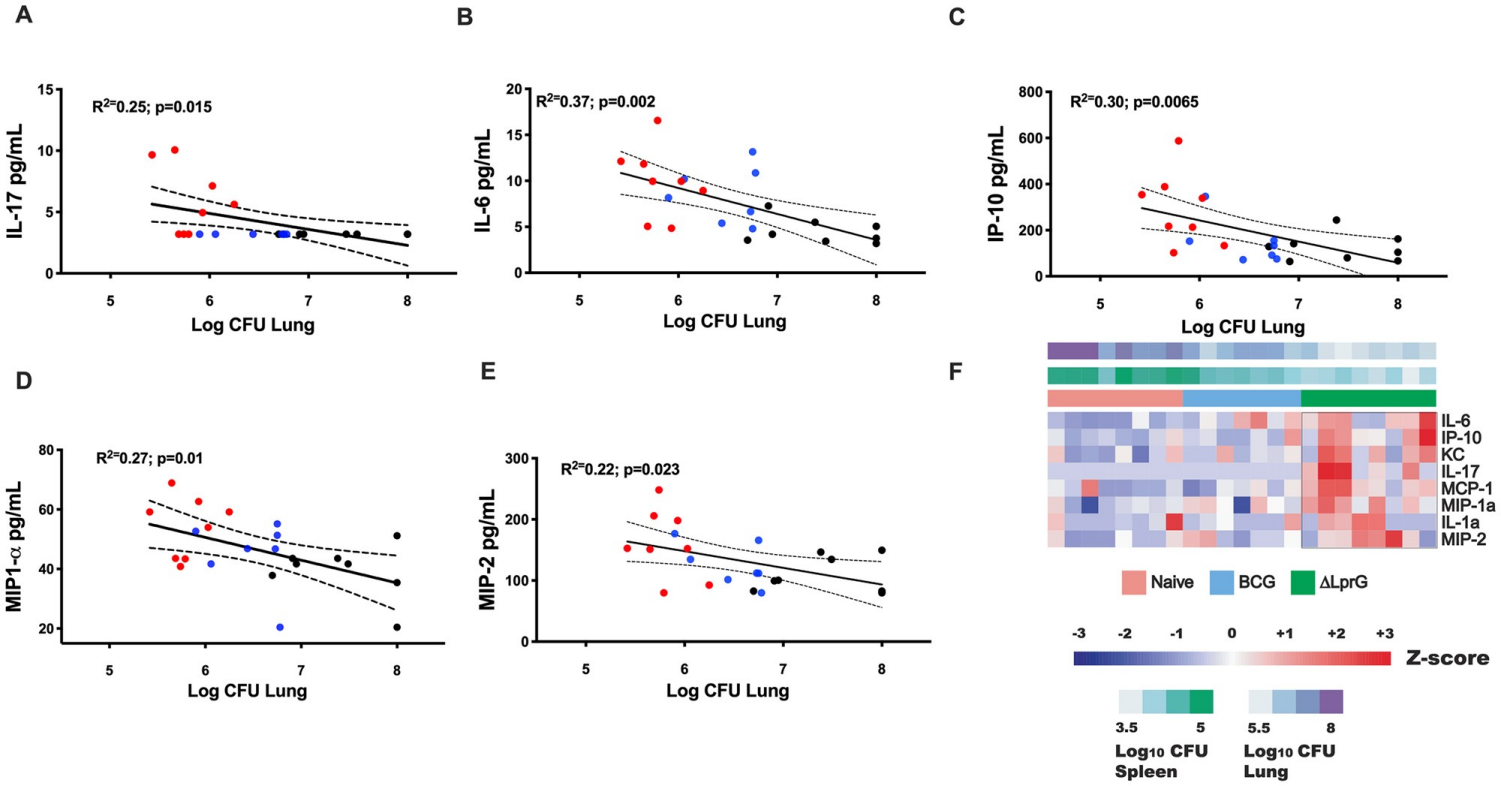
Correlations of serum cytokine levels following vaccination with CFU in lung following *Mtb* challenge. Serum cytokine levels from naïve and vaccinated mice were assessed at week 2 after vaccination by Luminex assays. Mice were challenged with 75 CFU *Mtb*. Pearson’s correlations of cytokine levels with CFU in lung following challenge for **A)** IL-17 **B)** IL-6 **C)** IP-10 **D)** MIP1-α and **E)** MIP-2. Red = ΔLprG vaccinated; Blue = BCG vaccinated; Black = Naïve mice. **F)** Heatmap of the normalized cytokines serum levels using z-score approach, correlated with Log10 CFU in lung (purple) and spleen (green) two weeks post-vaccination in Naïve (coral), BCG (light blue), and ΔLprG (green) vaccinated mice. CFU levels were used as a continuous variable. Each column represents an individual animal and each row represents an individual cytokine. Z-score levels range from blue (negatively correlated with CFU) to red (positively correlated with CFU). Serum cytokine correlation analyses are representative of two experiments with 8–10 mice per group. See also S2 Table.

We then asked whether we could use serum cytokine screening to detect early induction of a Th17 cytokine signature during peak immunogenicity post-vaccination, thereby functioning as an early screen for vaccine efficacy in C3HeB/FeJ mice. Consistent with these data, serum IL-17A at week 2 following vaccination was elevated in ΔLprG vaccinated mice as compared to naïve and BCG-vaccinated mice (Fig 6C, p = 0.0053 and 0.0053, respectively). Serum IL-17A levels at week 2 following vaccination correlated with IL-17A-secreting CD4^+^ T cells in lungs following challenge (S1 Table). Indeed, elevated IL-17A levels in serum 2 weeks post-vaccination significantly correlated with reduced lung bacterial burden 4 weeks post-*Mtb* challenge (Fig 7A; p = 0.015). IL-6, IL-1β, and IL-23 have also been reported as Th17 polarizing cytokines [27]. IL-6, MIP-1α, MIP-2, and IP-10, were also elevated in BCG and ΔLprG vaccinated mice (S5 Fig) and individually correlated with decreased bacterial burden in lungs following challenge (Fig 7B–7E; p = 0.002 to p = 0.02). To determine if a combination of cytokines would be a better correlate of protection, we used a multiple linear regression analysis to develop a serum cytokine signature that included IL-17A, IL-6, MIP-1α and IP-10a, which correlated with CFU levels in both lung (p = 0.008) and in spleen (p = 0.02) following challenge (Fig 7F). However, this combined cytokine signature did not outperform individual cytokines alone, specifically IL-6 and IL-17A, in correlating with protection (S2 Table).

**Fig 8:**
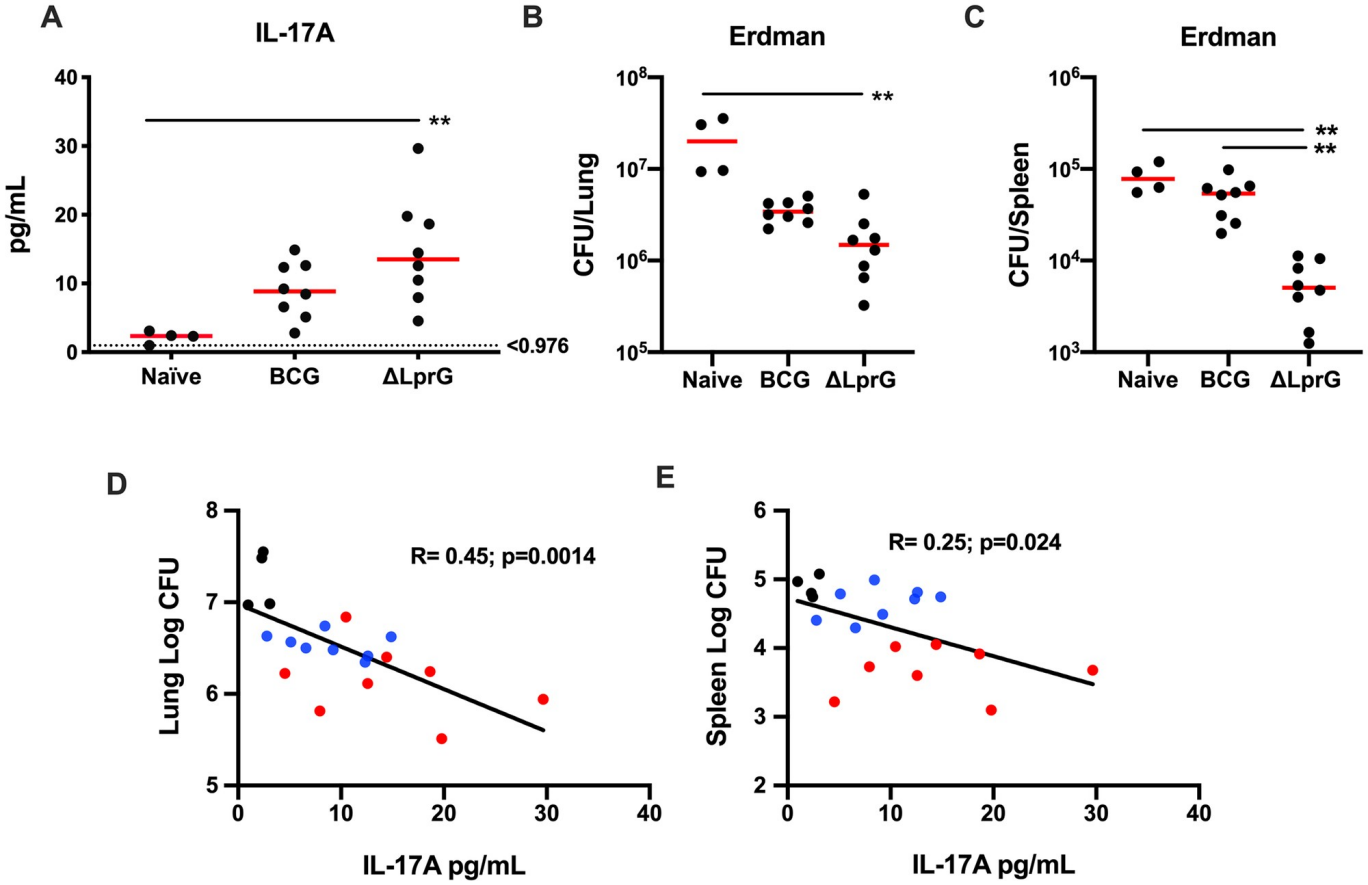
Validation of IL-17 serum immune correlate using high-sensitivity Luminex and *Mtb* Erdman challenge. Serum was collected from mice at week 2 following vaccination with BCG or ΔLprG as well as from naïve mice. **(A)** Serum IL-17A levels were evaluated using a high-sensitivity Luminex assay. Kruskall-Wallis with Dunn’s corrections for multiple comparisons; **p<0.01. Lower limit of quantification = 0.976 pg/mL. Red lines reflect median values. Log CFU in lung **(B)** and spleen **(C)** in C3HeB/FeJ in unvaccinated (Naïve) mice and in BCG or ΔLprG vaccinated mice at week 4 following aerosol challenge with 75 CFU *Mtb* Erdman. Pearson’s correlation of lung **(D)** and spleen **(E)** Log CFU following challenge with serum IL-17A levels following vaccination. Red = ΔLprG vaccinated; Blue = BCG vaccinated; Black = Naïve mice. Validation of serum IL-17 correlation with CFU following Erdman challenge was performed once with 4–8 mice per group. See also S7 Fig.

We next assessed the protective efficacy of the ΔLprG vaccine and the predictive capacity of serum IL-17A levels against challenge with the *Mtb* Erdman strain. In animal models such as the rabbit which demonstrate necrotizing granulomas with *Mtb* infection, Erdman is considered more pathogenic [33], and thus *Mtb* Erdman is generally considered a stringent challenge strain for vaccine studies. Furthermore, the genetic deletion of the LprG-Rv1410 operon was on the H37Rv background, and we wanted to test efficacy with a challenge strain different from that used to generate the ΔLprG vaccine. We vaccinated mice as described above and used a high sensitivity IL-17A Luminex panel to better quantify IL-17A levels in sera at week 2 following vaccination. Mice were then challenged with the *Mtb* Erdman strain. ΔLprG and BCG vaccination led to consistent induction of serum IL-17A two weeks following vaccination (Fig 8A), but ΔLprG afforded greater protection against *Mtb* Erdman challenge with a median 1.1 log reduction in bacterial CFU in lung (Fig 8B; p = 0.001) and a median 1.2 log reduction in bacterial CFU in spleen (Fig 8C; p = 0.002) compared to unvaccinated controls. In contrast, BCG afforded only a modest reduction in CFU in lung and spleen in this stringent challenge model. Serum IL-17A levels at week 2 following vaccination inversely correlated with bacterial burden in both lung (Fig 7D, p = 0.0014) and spleen (Fig 7E, p = 0.024) with a ROC AUC between 0.97–1.0 (S6 Fig) [34]. In contrast, levels of IL-6 levels failed to correlate with protection against *Mtb* Erdman challenge (S7 Fig). These data suggest that serum IL-17A may be a useful early biomarker following vaccination to predict protective efficacy.

## Discussion

The ΔLprG vaccine is a novel whole cell vaccine based on an *Mtb* strain that has been genetically engineered to delete key virulence factors and potential immune evasion genes. Vaccination with ΔLprG afforded equivalent protection to BCG in traditional vaccine testing models, C57BL/6J and Balb/cJ mice, but showed enhanced protection compared to BCG in C3HeB/FeJ mice against both *Mtb* H37Rv and Erdman challenges. Protection was associated with PD-1-negative and Th17 CD4^+^ T cells in lung post-*Mtb* challenge, and also correlated with serum IL-17A at week 2 following vaccination. These data support a model for pulmonary Th17 cells in TB vaccine efficacy and suggest that the improved efficacy of the ΔLprG vaccine over BCG in C3HeB/FeJ mice in these studies may be linked to higher induction of these immune subsets. Furthermore, we show that serum IL-17 early after vaccination should be explored as a possible biomarker for vaccine protective efficacy.

Prior studies of whole cell vaccines have included different versions of recombinant BCG, such as AERAS-22 [21, 35], in addition to whole cell mycobacterial vaccines based on attenuated clinical *Mtb* strains such as Mt103 [7,36–40]. Increasingly, whole cell vaccines have outperformed subunit and viral vectored vaccines in pre-clinical models [39–42].

The recent success reported for protection with intravenous (IV) BCG in non-human primates is promising and potentially could be enhanced with attenuated *Mtb* vaccines [43].

Vaccination with ΔLprG resulted in consistent reduction in bacterial burden following challenge in C57BL/6J and Balb/cJ mice and in C3HeB/FeJ mice following both H37Rv and Erdman *Mtb* challenges, suggesting that further studies are warranted to explore this strain as a candidate attenuated whole cell *Mtb* vaccine. Preclinical efficacy of MTBVAC has been reported in mice and guinea pigs [44], and MTBVAC is the first live attenuated *Mtb* whole cell vaccine to enter human clinical trials [7], demonstrating that attenuated *Mtb* can be administered safely in humans. The World Health Organization has stipulated that attenuated *Mtb* used as a vaccine must have two independent mutations [45]. Both MTBVAC and ΔLprG have stable genetic deletions in two genes, and both are non-pathogenic in immune deficient mice [11,46]. However, the two genetic deletions in the ΔLprG vaccine are in the same operon and therefore additional mutations may be needed prior to testing in humans. Future studies should explore whether the protective efficacy of the ΔLprG vaccine can be further enhanced by BCG priming [5] or by combining with other promising vaccine candidates [4]. We speculate that the protection afforded by the ΔLprG whole cell vaccine may be analogous to the robust protection observed with *Mtb* reinfection in cynomolgus macaques [6].

C3HeB/FeJ mice develop necrotizing granulomas more consistent with human tuberculous disease. Even as early as four weeks post-infection, pathology in C3HeB/FeJ mice showed divergent pathology from C57BL/6J and Balb/cJ mice with enhanced necrosis and neutrophilic infiltrates. Granulomas from ΔLprG vaccinated mice contained fewer mycobacteria than granulomas from BCG vaccinated mice suggesting that ΔLprG vaccination enhanced the restrictive nature of individual granulomas leading to overall decreased bacterial burden in the lung. C3HeB/FeJ mice are a preferred mouse model for evaluating the ability of antibiotics to penetrate the tissue microenvironment in pre-clinical TB drug development [47], and have been shown to be a robust model for testing vaccine efficacy [48], yet are infrequently used to test candidate TB vaccines. This may be due to the fact that there is variability in BCG protection dependent on strain [49]. In this study, BCG SSI failed to induce the typical 1 log of protection in C3HeB/FeJ mice as seen in C57BL/6J and Balb/cJ mice. The relatively modest protection with BCG vaccination in C3HeB/FeJ mice in our studies may have been related to the *Mtb* challenge strains used or the higher infectious inoculum (75 CFU as compared to 25 CFU) as compared with other studies [48]. Furthermore, C3HeB/FeJ mice are H2-k restricted and fail to present several CD4^+^ T lymphocyte antigens typically studied in the context of TB vaccines such as Ag85B which can limit comparison to the existing literature. Our data supports the use of this model for evaluating candidate TB vaccines in that it provides valuable insight on the ability of vaccines to improve function and clearance of bacteria at the level of the granuloma.

Future studies should evaluate the efficacy of ΔLprG and other candidate vaccines in long-term protection studies in C3HeB/FeJ mice. Emerging data have shown the conservation of immunodominant T cell epitopes across *Mtb* strains and the potential negative impact of immunodominant T cell responses on vaccine efficacy [50–52]. It will therefore be important to test candidate TB vaccines in multiple mouse models, including those that limit expansion of immunodominant clones to traditional TB antigens. Use of H2-k restricted mice such as C3HeB/FeJ may potentially enhance the identification of important subdominant antigen-specific responses induced by whole cell vaccines.

Vaccination with ΔLprG resulted in polyfunctional polyclonal Ag-specific CD4^+^ T cells to a PPD antigen, which included Th17 cells that co-expressed IFN-γ, TNF-α, and IL-2, key cytokines that have been reported to contribute to control of *Mtb* [53–56]. However, numerous studies in humans have shown that induction of polyfunctional CD4^+^ T cells in peripheral blood and lung are insufficient to predict efficacy of candidate TB vaccines [57]. Although relatively small, higher percentages of Ag-specific IL-17A expressing T cell subpopulations were seen in lungs of ΔLprG vaccinated mice post-*Mtb* challenge. Interestingly, induction of Th17 subsets was low in BCG vaccinated C3HeB/FeJ mice post-challenge, and BCG protection in C3HeB/FeJ mice was lower in these studies as compared to other published work [28], which may reflect differences in BCG vaccine preparation or strain. Future work using prime-boost regimens may enhance our ability to assess in greater detail Th17 cells post-vaccination and the potential mechanistic role of IL-17 and vaccine-elicited Th17 cells in pulmonary control of *Mtb*.

TB infection in mice, as in other chronic diseases and cancer [58], has been shown to induce T cell exhaustion that is associated with high bacterial burden in lung parenchyma [59,60]. Moreover, the diminished efficacy of BCG in adult populations has been hypothesized to reflect the generation of persistent effector memory T cells that become functionally exhausted over time [23]. In the HIV vaccine field, variation in the exhaustion profiles of candidate vaccines can have significant downstream immunological effects that could impact vaccine efficacy [61]. Although PD-1 expression was low on antigen-specific T lymphocytes in PBMC post-vaccination in C3HeB/FeJ mice, we observed strong association of PD-1-negative Ag-specific CD4^+^ T cells with decreased bacterial burden in lungs of ΔLprG vaccinated mice *post* Mtb challenge. In contrast, PD-1-positive CD4^+^ T cells, cytokine and non-cytokine producing cells in lung post-challenge correlated inversely with protective efficacy. Interestingly, vaccinated C3HeB/FeJ mice had increased percentages of PD-1 negative Ag-specific CD4+ T lymphocyte responses in the lung post-*Mtb* challenge. Future work will be needed to assess total cell numbers in vaccinated animals. However, these data suggest that functional, non-exhausted CD4^+^ T cells may be critical for vaccine protection and that exhausted CD4^+^ T cells may have a detrimental effect.

We also observed that serum IL-17A levels induced shortly after vaccination correlated with protection in C3HeB/FeJ mice. IL-17 secretion has been linked to both gamma-delta T cells and ILC3 cells, both of which have been implicated in IL-17 mediated down-stream improvements in phagocytic capacity and antigen presentation [62,63]. At early timepoints post-vaccination, ΔLprG was shown to induce numerous cytokines and chemokines associated with monocyte recruitment and activation (IP-10, MIG, and MCP-1). Correlations of serum IL-17A and associated chemokines with bacterial burden may reflect the multifactorial role of IL-17 in modulating both innate and adaptive immune responses. Future studies should explore the mechanistic role of IL-17 in vaccine responses and protection, including the use of blocking antibodies or knock-out mice, as well as correlations with lung Th17 cells prior to challenge and the generalizability of the utility of IL-17 as a serum biomarker for vaccine protective efficacy.

In summary, vaccination with ΔLprG resulted in bacterial control in lung and spleen following *Mtb* H37Rv challenge in C57BL/6J, Balb/cJ, and C3HeB/FeJ mice and also provided protection with *Mtb* Erdman challenge in C3HeB/FeJ mice. Protection was significantly associated with the percentages of PD-1-negative Th1 and Th17 Ag-specific cell populations in the lungs of mice after challenge [27,64]. Moreover, serum IL-17A levels at week 2 following vaccination correlated with protective efficacy. Taken together, these data define key immunologic pathways that may contribute to protection against *Mtb* challenges in mice. Further evaluation of the ΔLprG vaccine and the use of IL-17 as a serum biomarker for vaccine protective efficacy is warranted.

## Materials and methods

### Ethics statement

All animal experiments were performed under an animal protocol approved by the Harvard University Institutional Animal Care and Use and Committee.

### Mice, immunizations, and *Mycobacterium tuberculosis* infections

Female 6–10-wk-old C57BL/6J, Balb/cJ, SCID, and C3HeB/FeJ (The Jackson Laboratory, Bar Harbor, ME) were housed under sterile conditions in an ABSL3 facility. Mycobacterial strains were grown in 7H9 with 10% (vol/vol) OADC (Middlebrook), 0.2% glycerol and Tween 80 and maintained at 37°C with shaking at 100rpm unless otherwise indicated. Mycobacterial strains for vaccines lots were prepared as previously described with minor modifications [19]. Cultures of Bacillus Calmette-Guérin (BCG)-Danish (BCG SSI) originally obtained from Statens Serum Institute (Copenhagen, Denmark, *gift from William R*. *Jacobs)* and *Mtb* H37Rv Δ*lprG-rv1410c* (ΔLprG; *Martinot et*. *al*. *2016*) vaccines were expanded in 7H9 with 10% OADC, 0.5% glycerol, and 0.05% Tyloxapol to a 100mL volume in roller bottles. Log phase cultures were pelleted by centrifugation at 2000 rcf for 15 min and washed with equal volume (1:1) of PBS with 0.05% Tyloxapol, re-centrifuged followed by wash with 50% volume (1:2) of PBS-0.05% Tyloxapol, then re-centrifuged and resuspended in 25% volume (1:4) of PBS-0.05% Tyloxapol with 15% glycerol. To remove clumps, 10 mL volumes of bacterial suspension were filtered twice, first through 40μm then 20μm vacuum filter units (Millipore). The optical density 600 (OD600) of a 1:10 dilution of the resultant suspension was measured and then the filtrate was back diluted to OD 1.0 or OD 5.0 with additional of PBS-0.05% Tyloxapol with 15% glycerol. Mice received immunizations of 100uL of OD 1.0 bacterial culture (2 +/- 1x10^7^ CFU/mL) in PBS-0.05% Tyloxapol with 15% glycerol subcutaneously in the left flank of either BCG SSI or ΔLprG. Some mice received H37Rv prepared in a similar fashion as a control for immunogenicity studies. For dose finding experiments, C57BL/6J mice were vaccinated with BCG Pasteur or ΔLprG from freshly propagated vaccine cultures. Mice were rested a minimum of 8 weeks post-vaccination prior to aerosol challenge with 75 +/-25 CFU of either *Mycobacterium tuberculosis* H37Rv or Erdman.

### Tissue processing and CFU enumeration

In all studies, lungs from *Mtb* challenged mice were aseptically collected 4 weeks following *Mtb* challenge and perfused with PBS prior to tissue harvest to remove red blood cells by transection of the abdominal aorta followed by injection of the right ventricle with 10mL cold sterile phosphate buffered saline. The three right lung lobes were used to enumerate CFU and were homogenized in sterile 1xPBS, followed by serial dilutions onto 7H10 plates and incubated for 3 weeks at 37°C. The left lung lobe was collected into RPMI with 10% fetal bovine serum (FBS) and homogenized using scissors for lung leukocyte isolation. Lung homogenate was incubated for 30 min in digestion buffer containing RPMI, FBS, Type IV collagenase (Sigma C5138) and DNase at 37°C with gentle rocking. Lung digests were filtered through 30μm MACS SmartStrainers (Miltenyi Biotec) and washed with fresh media and resuspended in a standardized volume of 6 mL media prior to plating in 96 well U-bottom plates at an approximate density of 2x10^6^ cells per well. In some cases, the accessory lobe was inflated with 100uL of 10% neutral buffered formalin for histopathology.

### Flow cytometry and intracellular cytokine staining (ICS)

Lymphocytes were isolated from either blood, spleen, or lung, stained, and analyzed by flow cytometry as previously described [65]. Antibodies (Ab) to CD8a (53–6.7), CD4 (RM4-5), CD44 (IM7), PD-1 (RMPI-30), IFN-γ (XMG1.2), TNF-α (MP6-XT22), IL-2 (JES6-5H4), IL-17 (TC11-18H10), and IL-10 (JES5-16E3) were purchased from BD Biosciences (Myrtle, U.K.), eBioscience, or BioLegend (San Diego, CA). Cell viability was assessed by LIVE/DEAD Fixable Aqua (Life Technologies). Ag-specific cells were estimated by intracellular cytokine staining (ICS). Peripheral blood mononuclear cells (PBMC), splenocytes, or lung leukocytes were stimulated with a 1:200 dilution of purified protein derivative (PPD, Tuberculin OT, Synbiotics, Corp, San Diego) and incubated for 40 min at 37°C. After this incubation, Golgi-Plug and Golgi-Stop (BD Biosciences) were added and samples incubated for an additional 6.5 hours at 37°C. Cells were subsequently washed and stained for surface antibody markers, then permeabilized with Cytofix/Cytoperm (BD Biosciences) and stained for intracellular cytokines. Cells were acquired on an LSR II flow cytometer (BD Biosciences). Data were analyzed using FlowJo v10.

### Luminex assays for cytokine secretion

Serum samples were collected at days 1, 8 (+/- 1 day), and 14 days (+/- 1 day) post vaccination and compared to non-vaccinated Naïve mice. Samples were filtered twice through 0.2 micron 96 well filtration plates by centrifugation (Millipore), treated with 0.05% Tween-20, and assayed using a Luminex bead-based multiplex ELISA (MILLIPLEX MAP Mouse Cytokine/Chemokine Magnetic Bead Panel (Millipore; MCYTMAG-70K-PX32) according to manufacturer’s instructions. Samples were fixed with 4% formaldehyde and subsequently washed prior to acquisition. Sample data were acquired on a MAGPIX instrument running xPONENT 4.2 software (Luminex Corp.) and analyzed using a five-parameter logistic model with an 80–120% standard acceptance range. The MILLIPLEX MAP Mouse High Sensitivity T cell panel (Millipore; MHSTCMAG-70K) was used to evaluate low levels of IL-17A. <LLOQ indicates lower limit of quantification for assay; extrapolated values below the LLOQ were evaluated at the LLOQ. Multiple regression analyses of cytokines serum levels with CFU in lung and spleen were performed using the R mixOmics package [66]. Heatmaps of serum cytokine and chemokine levels in lung and spleen were generated using R heatmap package.

### Histopathology and image analysis

Lungs from infected mice were inflated with 10% neutral buffered formalin, processed, embedded in paraffin, and sectioned for staining. Formalin-fixed paraffin embedded (FFPE) serial tissue sections were stained with hematoxylin and eosin (H&E) and Ziehl-Neelsen acid-fast stains. Scoring for percent lung affected and acid-fast staining per granuloma was performed by two independent veterinary pathologists. Slides were digitized and lung granuloma area (mm^2^) quantified using Aperio Imagescope (Leica Biosystems).

### Statistical analysis

Statistical analyses were performed using Prism 8.0 (GraphPad Software). Data were analyzed by the Kruskal–Wallis test with Dunn multiple comparison post-test (more than two groups) or the two-tailed Mann–Whitney U test (for two groups). Longitudinally acquired Luminex data were analyzed using a two-tailed Mann-Whitney U test for each time point; not corrected for multiple comparisons across time points. Receiver operator curves (ROC) were generated using the ROC to baseline method with IL-17 values from the ultrasensitive Luminex assay [34].

## Supporting information

S1 Fig Effect of LprG-Rv1410 mutation on host recognition of T cell epitopes. C57BL/6J mice were injected subcutaneously with two doses of 100uL OD600 = 1.0 stocks of either BCG, *H37Rv*::*Δrv1411c-rv1410c*, or *H37Rv* and splenocytes were harvested 9 days post-boost. Percent cytokine positive CD4^+^ and CD8^+^ CD44^+^ antigen-specific splenocytes as measured by intracellular cytokine staining (ICS) following stimulation with 15-mer overlapping peptide pools spanning the entire Ag85B, ESAT-6, and TB10.4 proteins. Percentages reflect subsets of cytokine secreting cell populations from Boolean analysis (FlowJo v10) of all possible cytokine combinations (IFNγ, TNF-α, IL-2, IL-17A, and IL-10). Data representative of a single experiment with 5–8 animals per group.
(TIF)

S2 Fig Dose titration and protective efficacy of BCG Pasteur and ΔLprG vaccines in C57BL/6J mice. Female 6–8 wk old C57BL/6J mice were immunized subcutaneously with either 1x10^6^ or 1x10^7^ of freshly propagated vaccine cultures 8 weeks prior to aerosol challenge with 75 CFU of H37Rv *Mtb*. Lungs were homogenized and CFU enumerated 4 weeks post-challenge after growth on Middlebrook 7H10 agar. Data represents a single experiment performed once with 5 mice per group.
(TIF)

S3 Fig Cytokine secretion in splenocytes from naïve and vaccinated mice following *Mtb* challenge. Naïve, BCG, or ΔLprG vaccinated mice were challenged with 75 CFU *Mtb* H37Rv. Splenocytes were collected and stimulated with PPD. Percent cytokine secreting **(A)** CD4^+^ and **(B)** CD8^+^ CD44^+^ T cells are shown. Data representative of one of two experimental replicates with 5 mice per group.
(TIF)

S4 Fig PD-1-negative CD4^+^ T cell responses in naïve and vaccinated C3HeB/FeJ mice following *Mtb* challenge. Naïve, BCG, or ΔLprG vaccinated mice were challenged with 75 CFU *Mtb* H37Rv. T cells from lung were collected and stimulated with PPD. PD-1-negative populations shown in each panel. % IFN-γ, TNF-α, IL-2 positive PD-1^-^ CD4^+^ T cells are shown. Kruskall-Wallis with Dunn’s corrections for multiple comparisons;* p<0.05; ** p<0.01; *** p<0.001. Red bar indicates median values. Data representative of 2 experimental replicates with 5–8 mice per group.
(TIF)

S5 Fig Serum cytokine secretion in naïve and vaccinated mice at week 2 following vaccination. **A)** Heatmap of median log2 fold-change cytokine and chemokine secretion in sera from BCG and ΔLprG vaccinated mice as compared to naïve animals at week 2 following vaccination as measured by Luminex assays. **B-E)** Serum cytokine levels from naïve and vaccinated mice. Bars represent median values. LLOQ represents lower limit of quantification. Data representative of two experimental replicates with 5 mice per group.
(TIF)

S6 Fig ROC curve for high-sensitivity IL-17A serum Luminex assay. Mice were vaccinated with BCG and ΔLprG as described and sera collected for Luminex. The x-axis represents baseline percentiles and the y-axis is the probability that the BCG vaccinated values (filled squares) or ΔLprG vaccinated values (filled circles) were greater than or equal to the baseline percentile threshold as calculated using Graphpad prism v8. Data presents a single experiment performed once with 4–8 mice per group.
(TIF)

S7 Fig Serum IL-6 levels in vaccinated mice challenged with *Mtb* Erdman. IL-6 cytokine levels in sera from BCG and ΔLprG vaccinated mice as compared to naïve animals at week 2 following vaccination as measured by Luminex assays. Correlations of serum IL-6 levels lung and spleen CFU in mice challenged with *Mtb* Erdman four weeks post-challenge. *Mtb* Erdman challenge was performed once with 4–8 mice per group.
(TIF)

S1 Table Correlations of %IL-17A+ CD4+ T cells in lung with post-vaccination serum IL-17A levels and lung bacterial burden. Naïve, BCG, or ΔLprG vaccinated mice were challenged with 75 CFU *Mtb* H37Rv. Serum cytokine levels from naïve and vaccinated mice were assessed at week 2 after vaccination by Luminex assays. Mice were challenged with 75 CFU *Mtb* H37Rv. Mice were sacrificed at week 4 following challenge and perfused with sterile saline prior to tissue harvest. T cells in lung were collected and stimulated with PPD. Pearson’s correlations of % cytokine^+^ CD4^+^ T cells with serum IL-17A levels following vaccination and with CFU in lung are shown, and p-values.
(EPS)

S2 Table Correlations of serum cytokine levels following vaccination with CFU following challenge. P values of correlations of serum cytokine and chemokine levels at week 2 following vaccination with CFU levels in lung and spleen following challenge.
(EPS)
