# Supplementary figures and images for: Protective efficacy of an attenuated Mtb ΔLprG vaccine in mice
Source: PLoS Pathog. 2020 Dec 14;16(12):e1009096. doi: 10.1371/journal.ppat.1009096 (PMC7769599; doi:10.1371/journal.ppat.1009096)

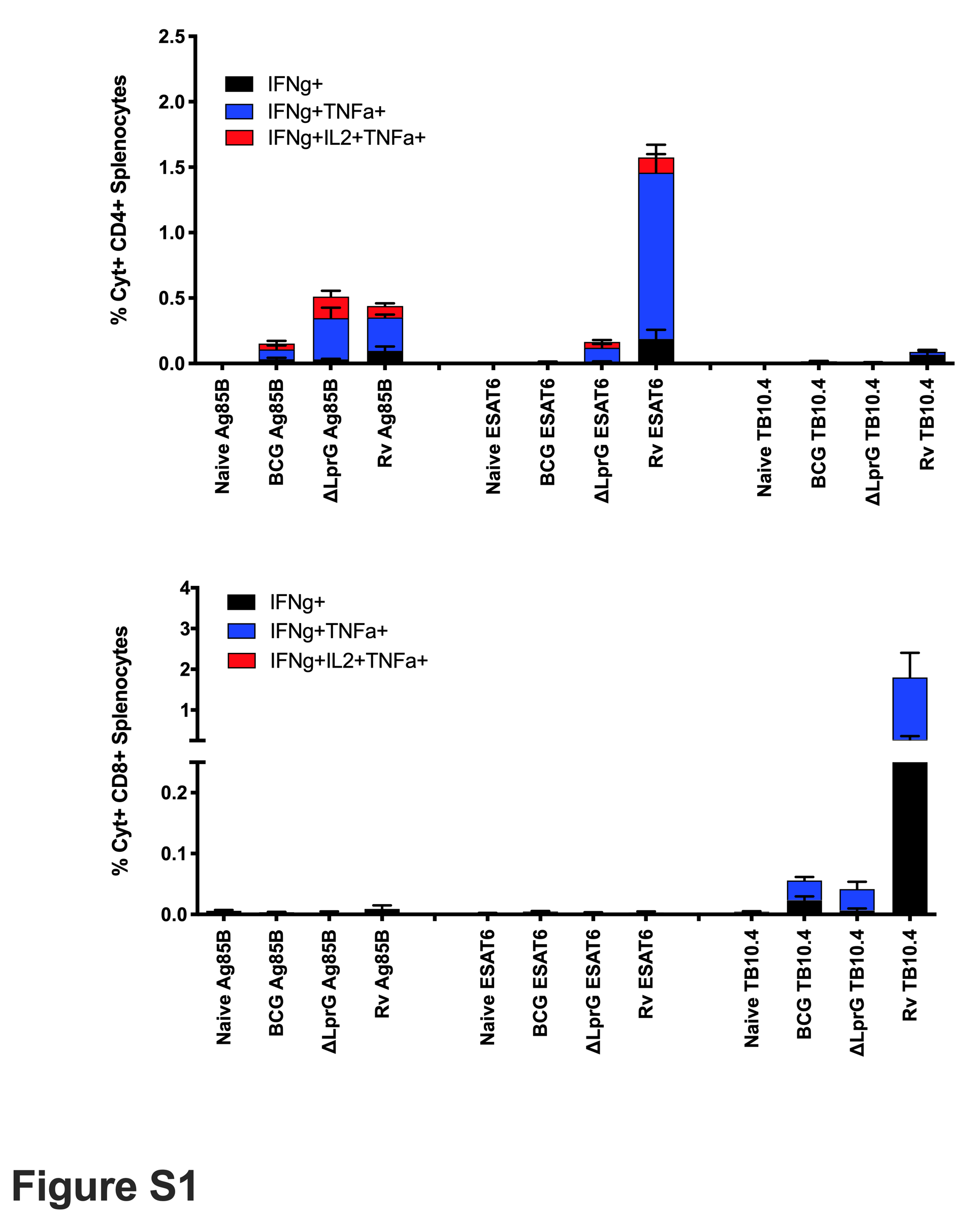

Supplement: S1 Fig — C57BL/6J mice were injected subcutaneously with two doses of 100uL OD600 = 1.0 stocks of either BCG, H37Rv::Δrv1411c-rv1410c, or H37Rv and splenocytes were harvested 9 days post-boost. Percent cytokine positive CD4+ and CD8+ CD44+ antigen-specific splenocytes as measured by intracellular cytokine staining (ICS) following stimulation with 15-mer overlapping peptide pools spanning the entire Ag85B, ESAT-6, and TB10.4 proteins. Percentages reflect subsets of cytokine secreting cell populations from Boolean analysis (FlowJo v10) of all possible cytokine combinations (IFNγ, TNF-α, IL-2, IL-17A, and IL-10). Data representative of a single experiment with 5–8 animals per group. (TIF) [file ppat.1009096.s001.tif]

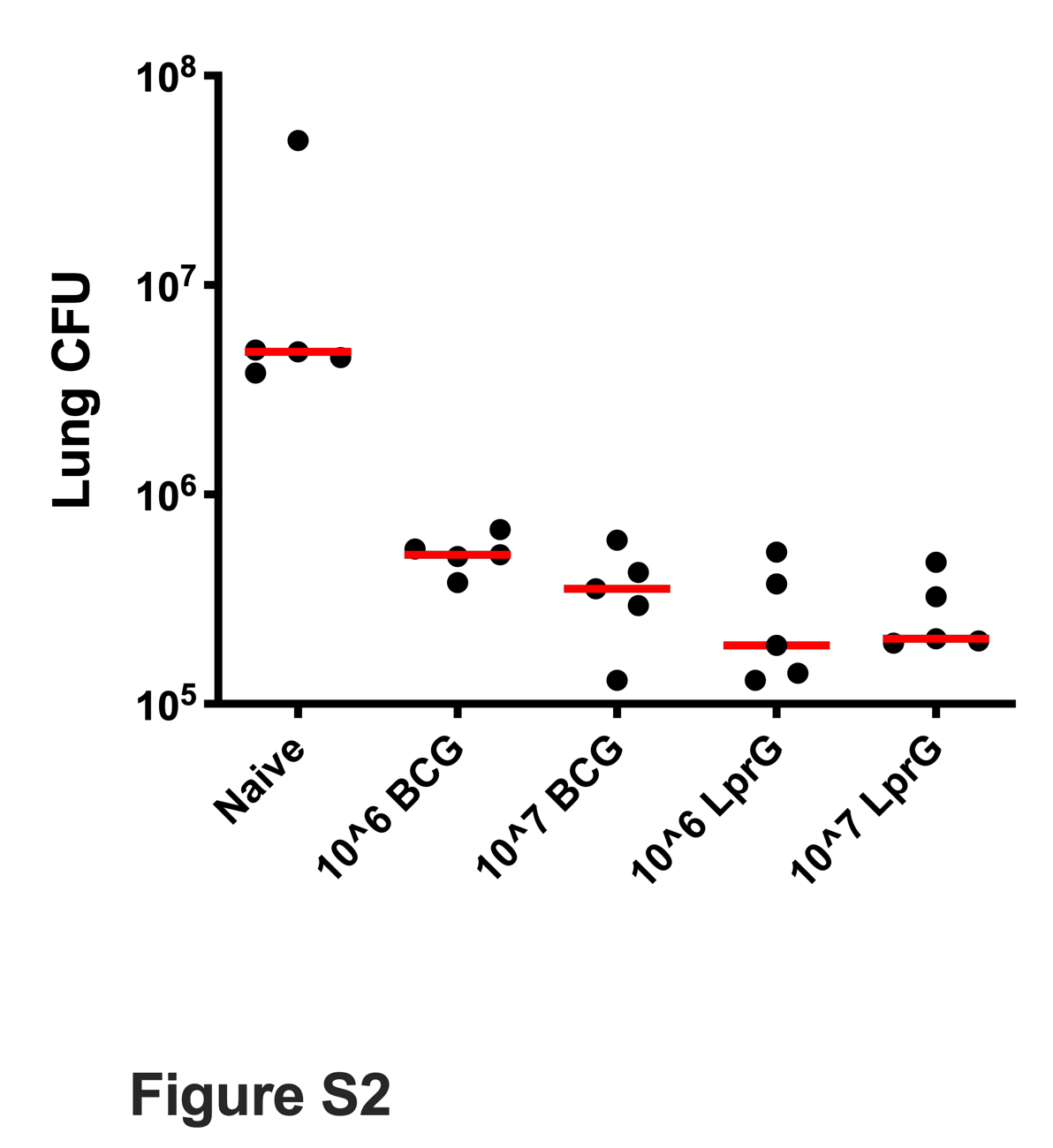

Supplement: S2 Fig — Female 6–8 wk old C57BL/6J mice were immunized subcutaneously with either 1x10^6^ or 1x10^7^ of freshly propagated vaccine cultures 8 weeks prior to aerosol challenge with 75 CFU of H37Rv Mtb. Lungs were homogenized and CFU enumerated 4 weeks post-challenge after growth on Middlebrook 7H10 agar. Data represents a single experiment performed once with 5 mice per group. (TIF) [file ppat.1009096.s002.tif]

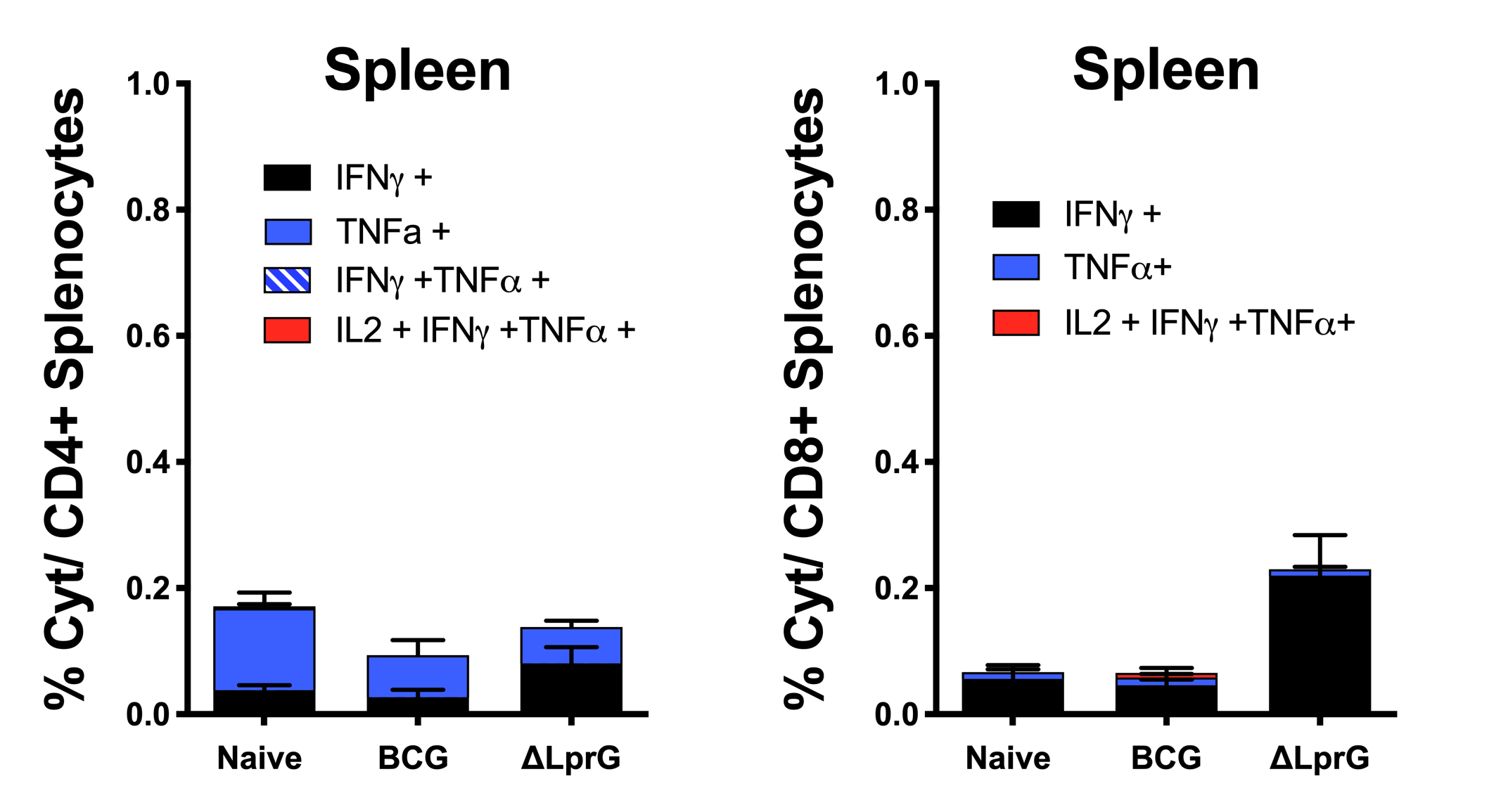

Supplement: S3 Fig — Naïve, BCG, or ΔLprG vaccinated mice were challenged with 75 CFU Mtb H37Rv. Splenocytes were collected and stimulated with PPD. Percent cytokine secreting (A) CD4+ and (B) CD8+ CD44+ T cells are shown. Data representative of one of two experimental replicates with 5 mice per group. (TIF) [file ppat.1009096.s003.tif]

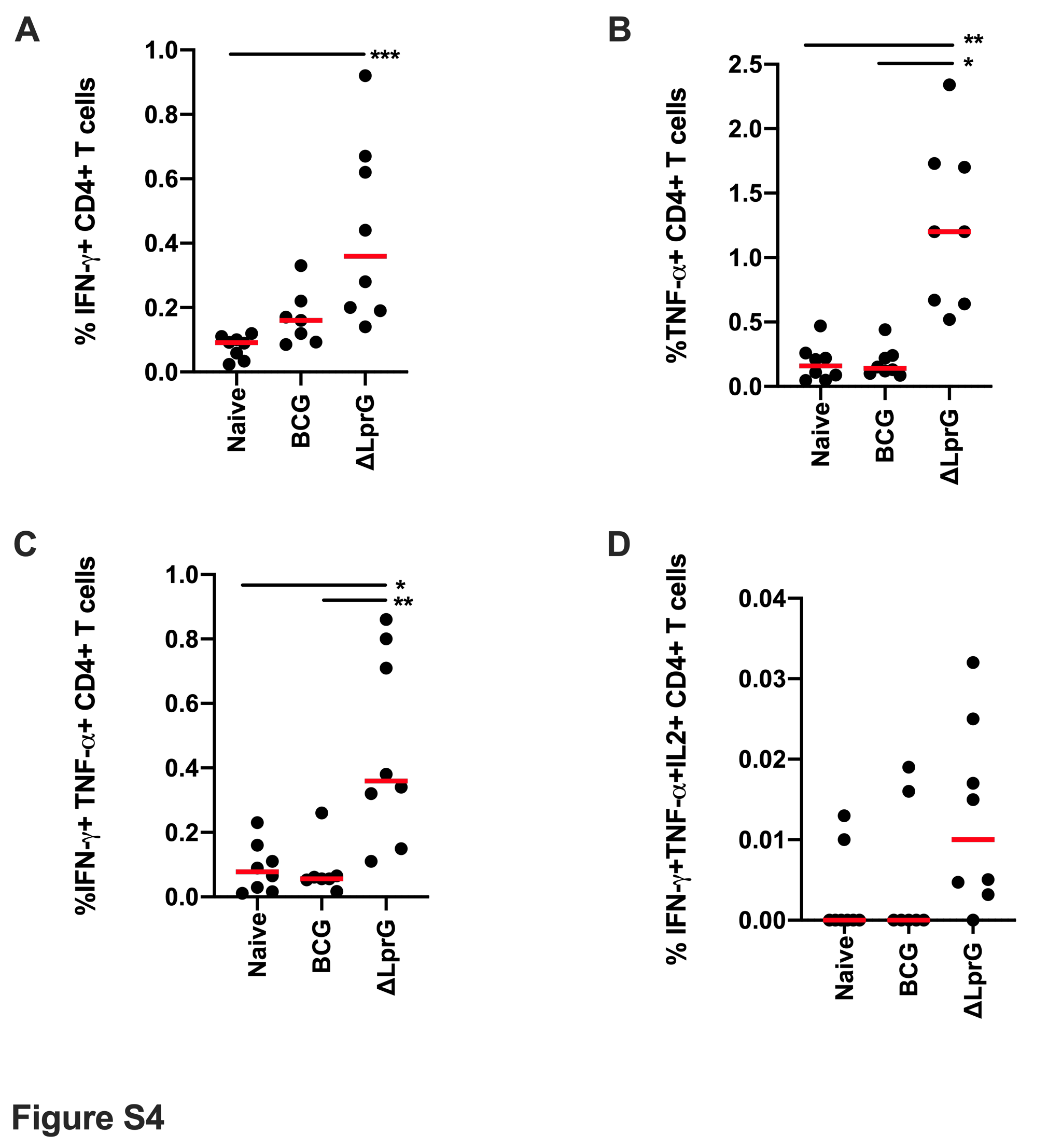

Supplement: S4 Fig — Naïve, BCG, or ΔLprG vaccinated mice were challenged with 75 CFU Mtb H37Rv. T cells from lung were collected and stimulated with PPD. PD-1-negative populations shown in each panel. % IFN-γ, TNF-α, IL-2 positive PD-1- CD4+ T cells are shown. Kruskall-Wallis with Dunn’s corrections for multiple comparisons;* p<0.05; ** p<0.01; *** p<0.001. Red bar indicates median values. Data representative of 2 experimental replicates with 5–8 mice per group. (TIF) [file ppat.1009096.s004.tif]

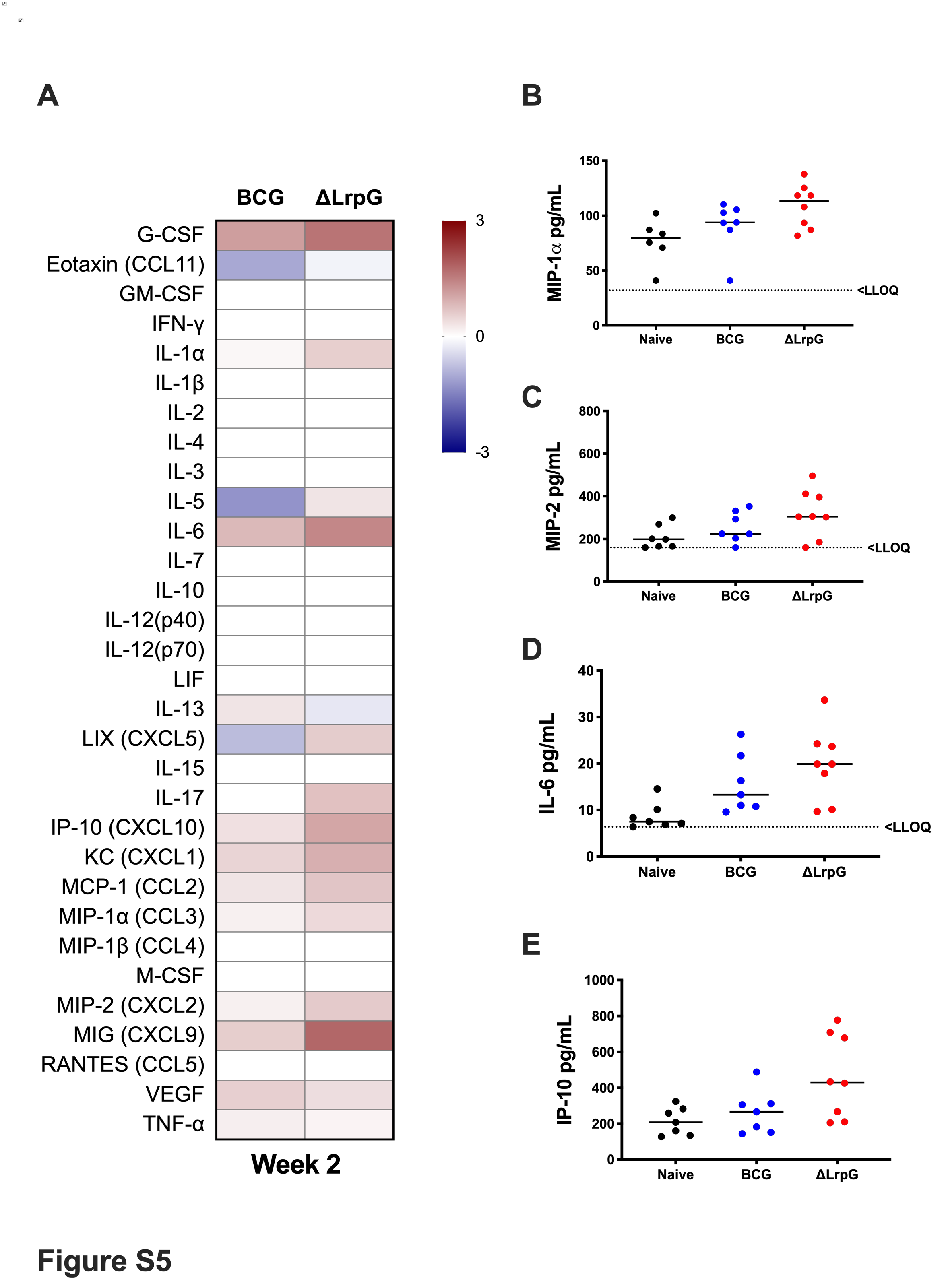

Supplement: S5 Fig — A) Heatmap of median log2 fold-change cytokine and chemokine secretion in sera from BCG and ΔLprG vaccinated mice as compared to naïve animals at week 2 following vaccination as measured by Luminex assays. B-E) Serum cytokine levels from naïve and vaccinated mice. Bars represent median values. LLOQ represents lower limit of quantification. Data representative of two experimental replicates with 5 mice per group. (TIF) [file ppat.1009096.s005.tif]

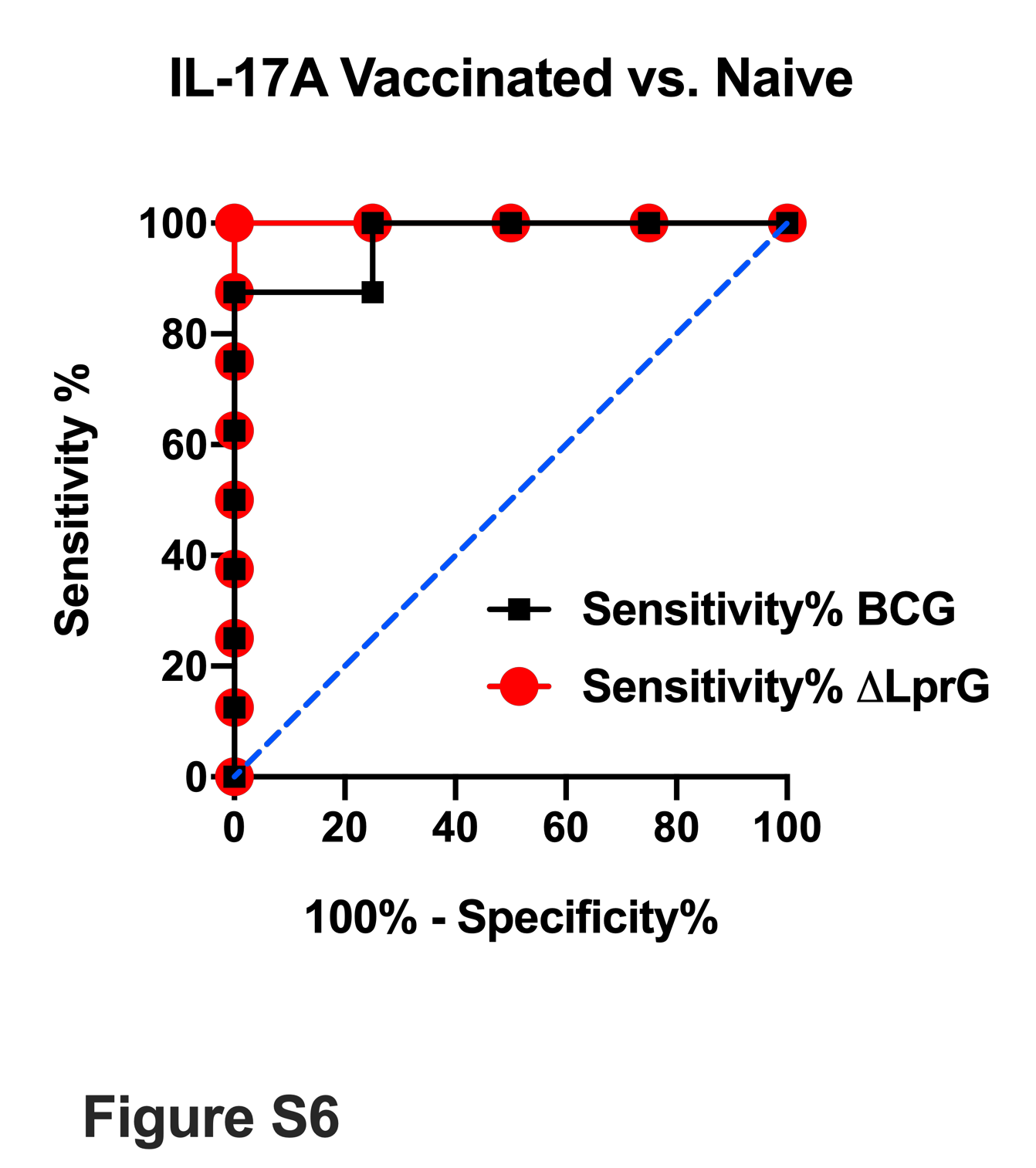

Supplement: S6 Fig — Mice were vaccinated with BCG and ΔLprG as described and sera collected for Luminex. The x-axis represents baseline percentiles and the y-axis is the probability that the BCG vaccinated values (filled squares) or ΔLprG vaccinated values (filled circles) were greater than or equal to the baseline percentile threshold as calculated using Graphpad prism v8. Data presents a single experiment performed once with 4–8 mice per group. (TIF) [file ppat.1009096.s006.tif]

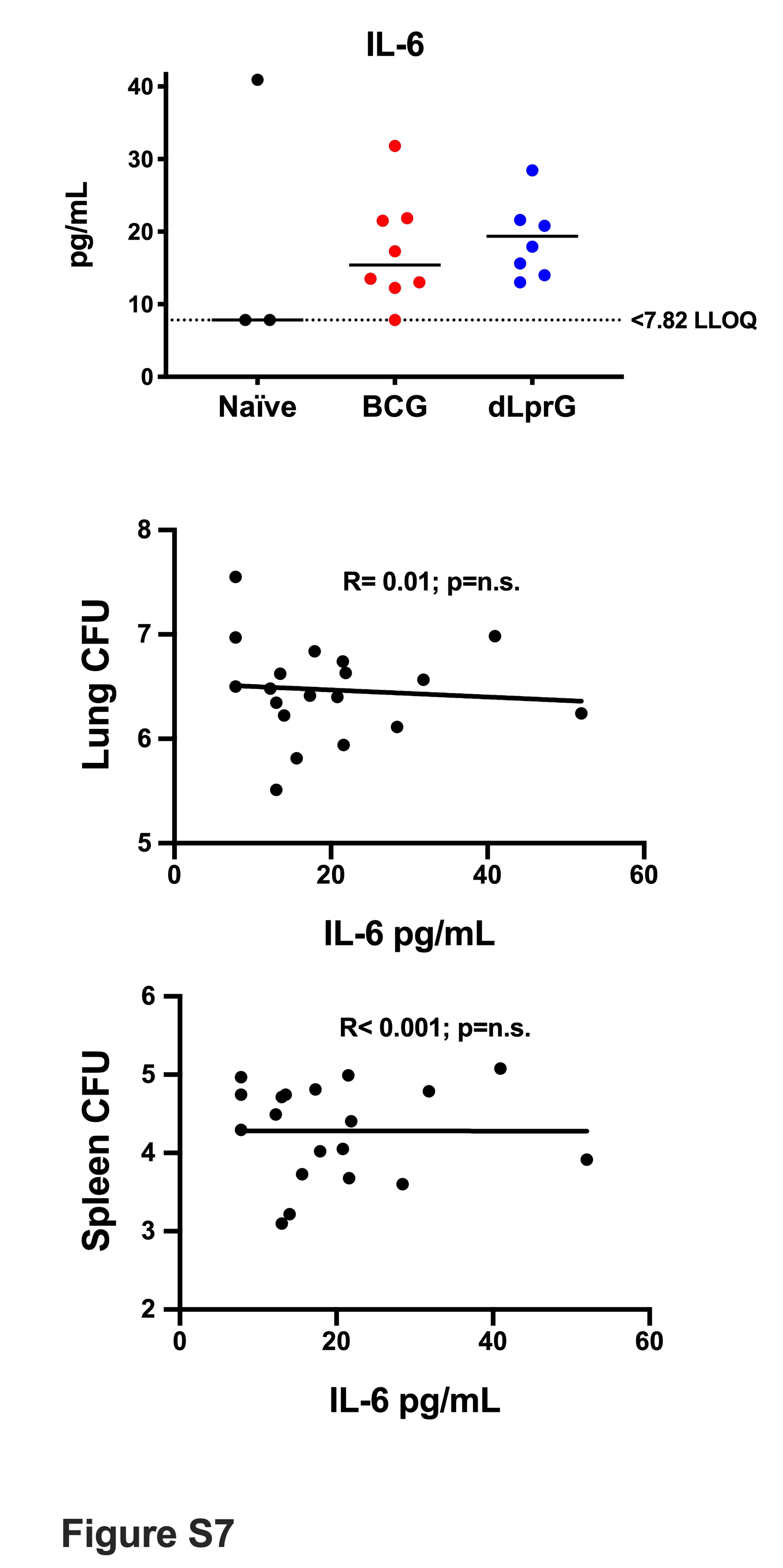

Supplement: S7 Fig — IL-6 cytokine levels in sera from BCG and ΔLprG vaccinated mice as compared to naïve animals at week 2 following vaccination as measured by Luminex assays. Correlations of serum IL-6 levels lung and spleen CFU in mice challenged with Mtb Erdman four weeks post-challenge. Mtb Erdman challenge was performed once with 4–8 mice per group. (TIF) [file ppat.1009096.s007.tif]
